# SCF^β-TrCP^-mediated degradation of TOP2β promotes cancer cell survival in response to chemotherapeutic drugs targeting topoisomerase II

**DOI:** 10.1038/s41389-020-0196-1

**Published:** 2020-02-03

**Authors:** Jianfeng Shu, Danrui Cui, Ying Ma, Xiufang Xiong, Yi Sun, Yongchao Zhao

**Affiliations:** 1 Key Laboratory of Combined Multi-Organ Transplantation, Ministry of Public Health, First Affiliated Hospital, Zhejiang University School of Medicine, Hangzhou, China; 2 Institute of Translational Medicine, Zhejiang University School of Medicine, Hangzhou, China; 3 Cancer Institute of the Second Affiliated Hospital, Zhejiang University School of Medicine, Hangzhou, China

**Keywords:** Chemotherapy, Ubiquitylation

## Abstract

Topoisomerase II (TOP2)-targeting anticancer chemotherapeutic drugs, termed TOP2 poisons, are widely used and effective in the clinic by stabilizing TOP2-DNA covalent complexes to induce DNA double-strand breaks (DSBs) and ultimately, cause cell death. The stabilized TOP2-DNA complex is known to be degraded by proteasome, whereas the underlying mechanism for instant TOP2β degradation in response to TOP2 poisons and the subsequent biological consequence remain elusive. Here, we reported that TOP2 poison-induced TOP2β degradation is mediated by SCF^β-TrCP^ ubiquitin ligase. Specifically, DNA damage signal, triggered by teniposide (VM-26) treatment, activates ATM, cooperating with CK1 to phosphorylate TOP2β on Ser1134 and Ser1130, respectively, in a canonical degron motif to facilitate β-TrCP binding and subsequent degradation. Inactivation of ATM, CK1 or SCF^β-TrCP^ by small molecular inhibitors or genetic knockdown/knockout abrogates TOP2β degradation. Biologically, blockage of TOP2β degradation in combination with VM-26 treatment impairs DNA damage response and repair, leading to an accelerated cell death via apoptosis. Thus, it appears that TOP2β degradation is a cellular defensive mechanism to facilitate the exposure of DSBs to trigger DNA damage response and repair. Collectively, our findings reveal a new strategy to improve the efficacy of TOP2 poisons in combination with small-molecule inhibitors against TOP2β degradation.

## Introduction

Type II DNA topoisomerase (TOP2) modulates DNA topology during DNA replication, transcription, repair, recombination, and chromosome remodeling and segregation^1–3^. TOP2 covalently binds to DNA, forming TOP2-DNA cleavable complexes, generates transient double-strand breaks (DSBs), which enables another duplex DNA to pass through the breaks, and finally, reseals the broken DNA strands^3,4^. Mammalian cells express two TOP2 isoforms, known as TOP2α and TOP2β. These two TOP2 isozymes share ~70% sequence identity and have similar catalytic activities and structural features but play distinct roles in biological processes^1,3^. TOP2α is primarily involved in regulating cell proliferation, whereas TOP2β is mainly associated with cell differentiation and transcription^5^. Both TOP2 isozymes have been shown to be cellular targets by numerous TOP2-targeting anticancer drugs in clinical use^6,7^.

A class of anticancer drugs that target TOP2, termed TOP2 poisons, including etoposide (VP-16)^8^, teniposide (VM-26)^6^, doxorubicin (DOX)^9^, and mitoxantrone^10^, are widely used and rather effective in clinical cancer treatment. TOP2 poisons stabilize TOP2-DNA complexes to disrupt TOP2-mediated religation of the broken strands, which blocks DNA replication and transcription and induces DNA DSBs and ultimately causes cell death^7^. However, to repair DSBs induced by TOP2 poisons, cancer cells have developed mechanisms by which TOP2 is removed from the TOP2-DNA adducts to expose TOP2-concealed DNA DSBs, thereby triggering DNA damage response and DNA repair, leading to enhanced survival^7,11^. Previous studies have shown that TOP2 poisons lead to TOP2 degradation in a ubiquitin-proteasome dependent manner^12–15^, and TOP2β is preferentially degraded over TOP2α^14^. However, which E3 ligase is responsible for TOP2β ubiquitination and degradation in response to TOP2 poisons, and what is the biological consequence of TOP2β degradation, are previously unknown.

Cullin-RING ligase E3 ubiquitin ligases (CRLs) are the largest family of E3 ubiquitin ligases and are responsible for the ubiquitination of approximately 20% of cellular proteins that are degraded through the ubiquitin-proteasome system^16^. Of all CRL ligases, CRL1, also known as SCF (SKP1-Cullin 1-F box protein), is the best studied member, in which Cullin 1 (CUL1) acts as a scaffold protein to bring together other components, including the adaptor protein SKP1, the substrate recognition receptor F-box protein, and the RING subunit RBX1^16^. β-TrCP, one of well-established F-box proteins that recognize and specifically bind to their substrates, promotes the polyubiquitination and degradation of many key signal proteins, such as CDC25A, WEE1, IκBα, β-catenin, DEPTOR. Thus, β-TrCP plays a critical role in the regulation of a vast array of cellular processes, including cell cycle regulation, signal transduction, gene transcription and translation, apoptosis, migration, etc.^17–19^ However, whether and how SCF^β-TrCP^ ubiquitin ligase regulates DNA damage response and DNA repair by modulating TOP2β levels is previously unknown.

In this study, we demonstrated that VM-26-induced TOP2β degradation is mediated by SCF^β-TrCP^ E3 ubiquitin ligase. Upon VM-26 stimulation, CK1 and activated ATM mediate TOP2β phosphorylation at Ser1130 and Ser1134, respectively, within its consensus β-TrCP degron motif, which facilitates β-TrCP-TOP2β binding and subsequent polyubiquitination for targeted degradation. Inactivating β-TrCP via genetic depletion and pharmacological approaches not only extends the protein half-life of TOP2β but also impairs DNA damage response. Consequently, depletion of β-TrCP or ectopic expression of β-TrCP-resistant TOP2β mutants sensitizes cancer cells to VM-26 by promoting cell apoptosis. Our study provides a new strategy to improve the efficacy of TOP2-targeted anticancer drugs by combination with small-molecule inhibitors against TOP2β degradation.

## Results

### TOP2β is a novel substrate of CRL E3 ligases

Several studies have demonstrated that TOP2 poisons induce TOP2β degradation via the 26S proteasome^12–15^. However, the mechanism by which TOP2β protein stability is regulated in vivo remains poorly understood. Consistent with previous studies, we also found that the proteasome inhibitor MG132 caused an accumulation of TOP2β protein in a time-dependent manner (Fig. S1A). TOP2 poisons, including VP-16, VM-26, m-AMSA, and DOX, indeed induced significant degradation of TOP2β with minimal, if any, effect on TOP2α (Fig. S1B). Moreover, MG132 blocked VM-26-induced TOP2β degradation, indicating 26S proteasome-mediated degradation (Fig. S1C).

**Fig. 1:**
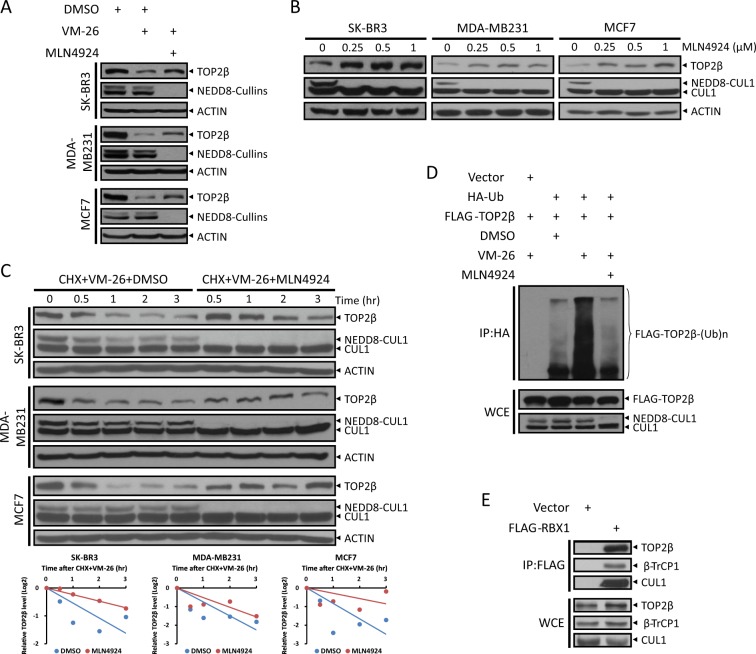
TOP2β is a novel substrate of CRL E3 ligases. **a** MLN4924 treatment blocks VM-26-induced TOP2β degradation. Cells were left untreated or treated with VM-26 (100 μM) or in combination with MLN4924 (1 μM) for 2 h. Cells were then harvested, and immunoblotting (IB) was undertaken using the indicated antibodies (Abs). **b** MLN4924 increases TOP2β levels in a dose-dependent manner. Cells were treated with various concentrations of MLN4924 for 24 h, and then, IB was undertaken with the indicated Abs. **c** MLN4924 treatment extends the protein half-life of TOP2β upon VM-26 treatment. Cells were treated with CHX (100 μg/ml) and VM-26 (100 μM) or in combination with MLN4924 (1 μM) for the indicated time periods, and then, IB was undertaken with the indicated Abs (top). Densitometry quantification was performed with ImageJ, and the decay curves are shown (bottom). **d** MLN4924 treatment suppresses the polyubiquitination of TOP2β induced by VM-26 treatment. HEK293 cells transfected with the indicated plasmids were treated with VM-26 (100 μM) and MG132 (20 μM) or in combination with MLN4924 (1 μM) for 5 h, and then, IP was conducted with anti-HA beads (top), and direct IB was undertaken with the indicated Abs (bottom). WCE: whole-cell extract. **e** RBX1 binds to endogenous TOP2β. HEK293 cells transfected with the indicated plasmids were harvested for IP with anti-FLAG beads (top) and direct IB with the indicated Abs (bottom).

Given that CRLs are the largest family of E3 ubiquitin ligases, to determine whether CRL E3 ubiquitin ligases are involved in TOP2 poison-induced TOP2β degradation, we treated cells with VM-26 in combination with MLN4924, a small-molecule inhibitor of the NEDD8-activating enzyme (NAE) that inactivates cullin neddylation to block CRL activation^20^. We found that the downregulation of TOP2β levels upon VM-26 treatment was abrogated by MLN4924 in human breast cancer SK-BR3, MDA-MB231, and MCF7 cells (Fig. 1a). Moreover, MLN4924 caused a dose-dependent accumulation of TOP2β in all tested cells (Fig. 1b). Similar to MG132, MLN4924 also significantly extended the protein half-life of TOP2β upon VM-26 treatment (Fig. 1c and Fig. S1C). Consistently, the polyubiquitination of TOP2β induced by VM-26 was remarkably inhibited by MLN4924 treatment (Fig. 1d). To further demonstrate that CRLs are involved in TOP2β degradation, we overexpressed FLAG-RBX1, one of two RING components in CRL ligases^16^, in HEK293 cells and found that RBX1 bound to endogenous TOP2β (Fig. 1e). As expected, CUL1 and β-TrCP1, two components of CRL1 ligase, were also pulled down by RBX1 (Fig. 1e). Together, these results suggest that TOP2β is targeted by CRL E3 ubiquitin ligase for ubiquitination and degradation upon VM-26 treatment.

### β-TrCP binds to TOP2β and negatively regulates TOP2β levels

We next determined whether CRL1 is the E3 ligase that mediated VM-26-induced TOP2β degradation by measuring the protein half-life of TOP2β upon CUL1 knockdown via siRNA oligos and found that CUL1 silencing extended the protein half-life of TOP2β upon VM-26 treatment (Fig. S2A), suggesting that CRL1/SCF ubiquitin ligase is responsible for TOP2β degradation.

**Fig. 2:**
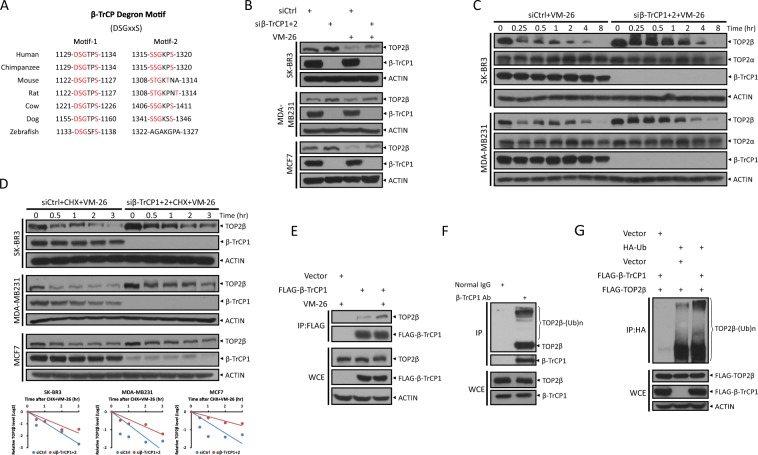
β-TrCP binds to TOP2β and negatively regulates TOP2β levels. **a** Evolutionary conservation of the β-TrCP degron motifs of TOP2β. **b** β-TrCP silencing abrogates TOP2β reduction by VM-26 treatment. Cells transfected with siRNA oligos targeting both β-TrCP1 and β-TrCP2 (siβ-TrCP1 + 2) or scrambled control siRNA (siCtrl) were left untreated or treated with VM-26 (100 μM) for 2 h, and then, IB was undertaken with the indicated Abs. **c** β-TrCP silencing suppresses the TOP2β degradation by VM-26 treatment in a time-dependent manner but has no effect on TOP2α. Cells transfected with siRNA oligos targeting β-TrCP or scrambled control siRNA were treated with VM-26 for the indicated time periods, and then, IB was undertaken with the indicated Abs. **d** β-TrCP silencing extends TOP2β protein half-life upon VM-26 treatment. Cells transfected with the indicated siRNA were treated with CHX and VM-26 for the indicated time periods and then subjected to IB with the indicated Abs. Densitometry quantification was performed with ImageJ, and the decay curves are shown (bottom). **e** Ectopically expressed β-TrCP1 binds to endogenous TOP2β. HEK293 cells transfected with the indicated plasmids were left untreated or treated with VM-26 and MG132 (20 μM) for 5 h. Cells were then harvested for IP with anti-FLAG beads (top) and direct IB with the indicated Abs (bottom). **f** β-TrCP1 binds to endogenous TOP2β. HeLa cells were lysed and subjected to IP with β-TrCP1 antibody or normal rabbit immunoglobulin (IgG), and then, IB was undertaken with anti-TOP2β Ab (top). Cell lysate was also directly immunoblotted with the indicated Abs (bottom). **g** β-TrCP1 promotes TOP2β polyubiquitination in vivo. HEK293 cells transfected with the indicated plasmids were treated with MG132 and VM-26 for 5 h, and then, IP was conducted with anti-HA beads (top), and direct IB was undertaken with the indicated Abs (bottom).

To further define which F-box protein specifically binds to TOP2β and targets it for ubiquitination, we examined the TOP2β protein sequence for the consensus binding motifs required for F-box protein binding, and identified two evolutionarily conserved putative binding motifs (^1129^DSGTPS^1134^ and ^1315^SSGKPS^1320^) for β-TrCP (DSGxxS) (Fig. 2a)^17^. Knocking down β-TrCP by siRNA oligos suppressed VM-26-induced TOP2β degradation (Fig. 2b) and caused a time-dependent accumulation of TOP2β with minimal, if any, effect on TOP2α (Fig. 2c). Consistently, β-TrCP silencing by siRNA oligos or by CRISPR-Cas9-mediated β-TrCP1 knockout significantly extended the protein half-life of TOP2β in all tested cells (Fig. 2d and Fig. S2B), suggesting that TOP2β degradation induced by VM-26 is mediated by β-TrCP. In addition, a co-immunoprecipitation assay using ectopically expressed FLAG-β-TrCP1 confirmed an interaction between β-TrCP1 and TOP2β, which was increased upon VM-26 treatment (Fig. 2e). Moreover, polyubiquitinated TOP2β can be readily detected in immune precipitates by β-TrCP1 (Fig. 2f). In addition, an in vivo ubiquitination assay further confirmed that β-TrCP1 markedly promoted TOP2β polyubiquitination induced by VM-26 treatment (Fig. 2g). Taken together, these results clearly demonstrate an interaction between TOP2β and β-TrCP1 in vivo and suggest that SCF^β-TrCP^ E3 ligase mediates ubiquitination and degradation of TOP2β induced by VM-26 treatment.

### Turnover of TOP2β is dependent on its β-TrCP degron motif

**Fig. 3:**
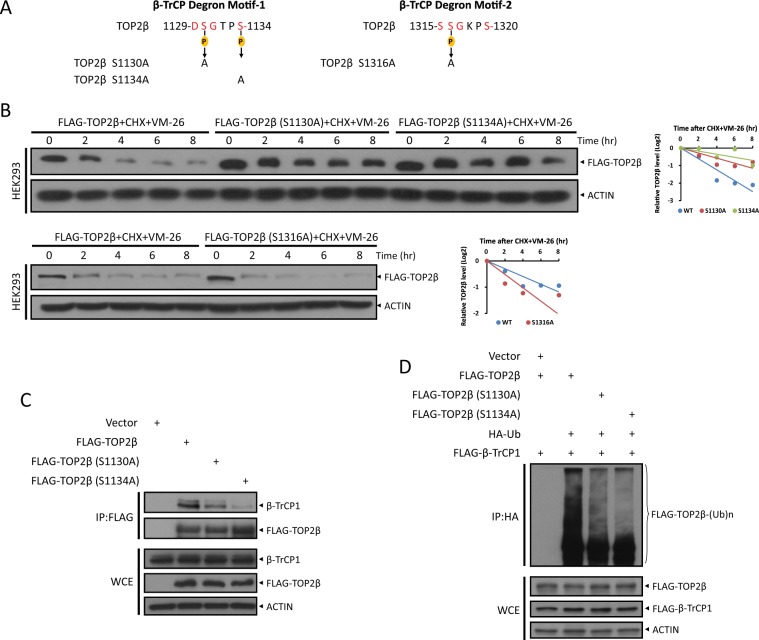
The turnover of TOP2β upon VM-26 treatment is dependent on the β-TrCP degron motif of TOP2β. **a** Diagram of mutants of two potential β-TrCP degron motifs in TOP2β. **b** TOP2β S1130A and S1134A mutants, but not the S1316A mutant, have longer protein half-lives. HEK293 cells transfected with wild-type or indicated mutants of FLAG-TOP2β were treated with CHX and VM-26 for the indicated time periods, and then, IB was undertaken using the indicated Abs (left). Densitometry quantification was performed with ImageJ, and the decay curves are shown (right). **c** Reduction in β-TrCP-TOP2β binding by degron site mutations. HEK293 cells transfected with the indicated plasmids were treated with MG132 and VM-26 for 5 h, and then, IP was conducted with anti-FLAG beads (top), and direct IB was undertaken with the indicated Abs (bottom). **d** Reduction in TOP2β ubiquitination by degron site mutations. HEK293 cells transfected with the indicated plasmids were treated with MG132 and VM-26 for 5 h, and then, IP was conducted using anti-HA beads (top), and direct IB was undertaken using the indicated Abs (bottom).

It is well established that the phosphorylation of the key residues on the degron motif is a prerequisite for the binding of a substrate to an F-box protein for subsequent ubiquitination and degradation by SCF ubiquitin ligases^21^. Specifically, the two serine residues of the β-TrCP degron motif (DSGxxS) must be phosphorylated. To determine whether the two putative β-TrCP degron motifs affected TOP2β-β-TrCP binding and TOP2β stability, we generated three TOP2β mutations in the β-TrCP degron motifs. Specifically, three serine residues were replaced with alanine, which cannot be phosphorylated: S1130A, S1134A, and S1316A (Fig. 3a). Immunoblotting assays revealed that the half-lives of proteins with the S1130A and S1134A mutations in degron motif-1 (^1129^DSGTPS^1134^) were obviously extended compared with that of wild-type TOP2β, whereas the S1316A mutation in degron motif-2 (^1315^SSGKPS^1320^) was not, but shortened the TOP2β protein half-life (Fig. 3b). These results indicated that β-TrCP degron motif-1 may be subjected to β-TrCP recognition and interaction. Indeed, an IP-based pull-down assay confirmed that S1130A and S1134A mutations disrupted the interaction between TOP2β and β-TrCP1 (Fig. 3c). Consequently, these two mutations in degron motif-1 significantly inhibited the polyubiquitination of TOP2β induced by VM-26 treatment in an in vivo ubiquitination assay (Fig. 3d). Thus, β-TrCP binds to and targets TOP2β for ubiquitination and degradation in a manner dependent on the phosphorylation of both serine residues in the β-TrCP degron motif-1, suggesting that TOP2β is a *bona fide* substrate of SCF^β-TrCP^ ubiquitin ligase.

### ATM binds to and phosphorylates TOP2β at Ser1134 to promote TOP2β degradation

**Fig. 4:**
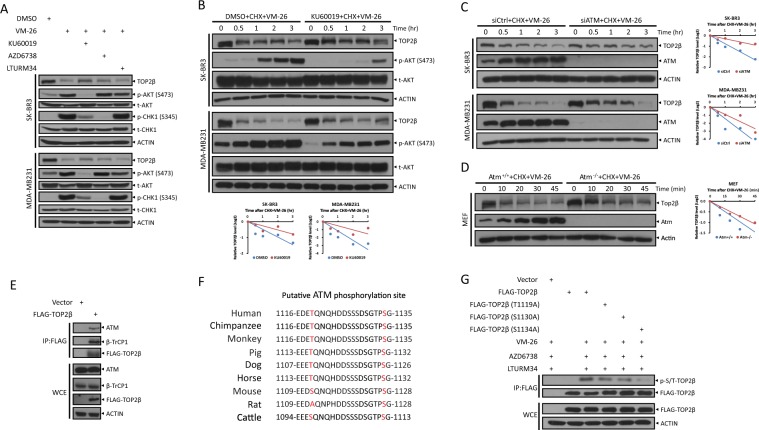
ATM binds with and phosphorylates TOP2β at Ser1134 to promote its degradation by VM-26. **a** Inhibition of ATM, but not ATR or DNA-PK, blocks VM-26-induced TOP2β degradation. Cells were pretreated with KU60019, AZD6738, or LTURM34 for 1 h and then treated with VM-26 for an additional 2 h. Cells were then harvested for IB with the indicated Abs. **b**–**d** Inhibition of ATM extends the protein half-life of TOP2β. SK-BR3 and MDA-MB231 cells were pretreated with DMSO or KU60019 (5 μM) for 1 h (**b**) or transfected with the indicated siRNA (**c**), followed by treatment with CHX and VM-26. Atm WT or KO MEFs (**d**) were also treated with CHX and VM-26 for various time periods, and then, IB was undertaken with the indicated Abs. Densitometry quantification was performed with ImageJ, and the decay curves are shown (bottom, **b**; right, **c** and **d**). **e** TOP2β binds to endogenous ATM. HEK293 cells were transfected with the indicated plasmids, and then, IP was conducted with anti-FLAG beads (top), and direct IB was undertaken with the indicated Abs (bottom). **f** Evolutionary conservation of two putative ATM phosphorylation sites (in red) on TOP2β is shown. **g** ATM phosphorylates TOP2β at Ser1134. HEK293 cells transfected with the indicated plasmids were left untreated or treated with VM-26, AZD6738, and LTURM34 for 5 h. Cells were harvested after MG132 treatment for 5 h, and then, IP was conducted using anti-FLAG Abs (top), and direct IB was undertaken with the indicated Abs (bottom).

It is well known that chemotherapeutic drugs targeting topoisomerases induce DNA damage^7^ and the three key kinases, including ATM, ATR, and DNA-PK, activated by DNA damage signals mediate DNA damage response to induce cell cycle arrest, DNA repair, and apoptosis^22^. To determine which VM-26-activated kinase (or kinases) mediates the phosphorylation of TOP2β at the serine residues of the consensus binding motif, thereby leading to its degradation, we used small-molecule inhibitors to inactivate ATM, ATR, or DNA-PK, respectively, and determined their effects on TOP2β protein levels. We found that VM-26-induced TOP2β reduction was significantly abolished by KU60019, an ATM inhibitor^23^, but not by the ATR inhibitor AZD6738^24^ or the DNA-PK inhibitor LTURM34^25^ (Fig. 4a). Consistently, VM-26, indeed, significantly activated ATM in a time-dependent manner, as reflected by the increase in the phosphorylation of ATM at Ser1981 (Fig. S3A). Moreover, the protein half-life of TOP2β was significantly extended in the presence of KU60019 but not AZD6738 or LTURM34 (Fig. 4b and Fig. S3B, C). More specifically, ATM knockdown via siRNA oligos in breast cancer SK-BR3 and MDA-MB231 cells (Fig. 4c) or ATM knockout in mouse embryonic fibroblasts (MEFs) (Fig. 4d) extended TOP2β protein half-life upon VM-26 treatment (Fig. 4c, d). In addition, ATM was readily detected in immune precipitates by FLAG-tagged TOP2β (Fig. 4e), indicating that ATM can bind to and phosphorylate TOP2β.

To identify which residue is phosphorylated by ATM, we examined the TOP2β protein sequence and found two putative ATM phosphorylation sites close to the consensus β-TrCP degron motif, Thr1119 and Ser1134, as predicted by computer-aided algorithms in GSP 2.0 (http://gps.biocuckoo.org) and KinasePhos 2.0^26^, respectively (Fig. 4f). Although both Ser/Thr residues are evolutionarily conserved (Fig. 4f), only the S1134A mutant significantly abrogated the phosphorylation of TOP2β, whereas the phosphorylation-dead mutations of Thr1119 or Ser1130, the other key serine residue in the β-TrCP degron motif, had no effects on the phosphorylation levels of TOP2β, as shown by the phosphorylation levels of immune precipitates by FLAG-tagged TOP2β using p-S/T antibody (Fig. 4g). Together, our results demonstrate that ATM binds to TOP2β and phosphorylates Ser1134 for targeted TOP2β degradation.

### CK1 phosphorylates TOP2β at Ser1130 to promote its degradation induced by VM-26

**Fig. 5:**
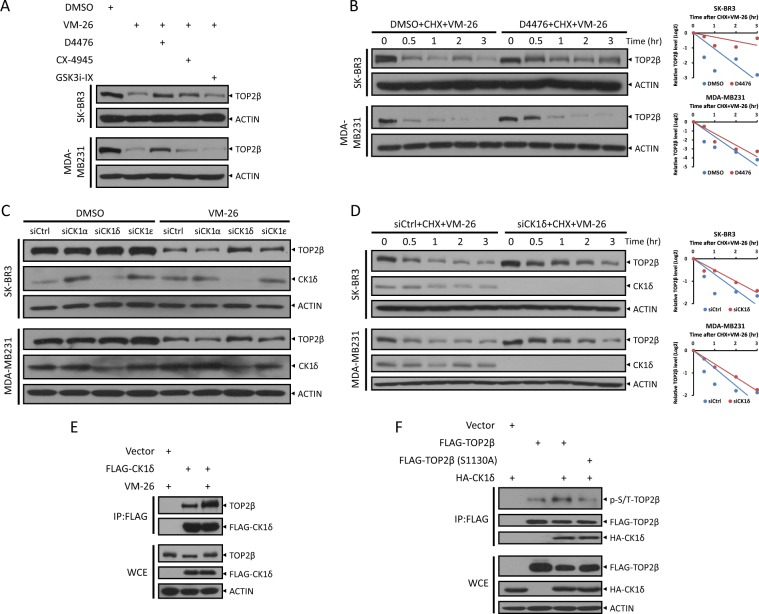
CK1 binds with and phosphorylates TOP2β at Ser1130 to promote its degradation by VM-26. **a** Inhibition of CK1, but not CK2 or GSK3, blocks the degradation of TOP2β induced by VM-26. Cells pretreated with D4476, CX-4945 or GSK3i-IX for 1 h were treated with VM-26 for an additional 2 h. Cells were then harvested for IB analysis with the indicated Abs. **b** Inhibition of CK1 extends the protein half-life of TOP2β. Cells left untreated or pretreated with D4476 (50 μM) for 4 h were then exposed to CHX and VM-26. Cells were harvested at the indicated time points for IB analysis with the indicated Abs (left). Densitometry quantification was performed with ImageJ, and the decay curves are shown (right). **c** and **d** Silencing of CK1 inhibits TOP2β degradation by extending its protein half-life. Cells transfected with the indicated siRNA oligos were treated with VM-26 for 1 h (**c**) or treated with VM-26 and CHX for the indicated time periods (**d**), and then, IB was undertaken with the indicated Abs. Densitometry quantification was performed with ImageJ, and the decay curves are shown (**d**, right). **e**, **f** CK1 binds to and phosphorylates TOP2β at Ser1130. HEK293 cells transfected with the indicated plasmids were treated with VM-26 for 5 h as indicated (**e**) or in combination with MG132 (**f**), and then, IP was conducted with anti-FLAG beads (top), and direct IB was undertaken with the indicated Abs (bottom).

Given that ATM is unable to phosphorylate TOP2β at Ser1130, which is essential for its degradation, we next explored other possible kinases involved in regulating TOP2β degradation with the focused on GSK3, CK1, and CK2, which often play roles in β-TrCP-mediated protein degradation^27^. Notably, inactivation of CK1 by D4476, a CK1 inhibitor^28^, blocked the degradation of TOP2β in response to VM-26 treatment, whereas inactivation of CK2 and GSK3 by CK2 inhibitor CX-4945^29^ and GSK3 inhibitor GSK3i-IX^30^, respectively, had no such effect (Fig. 5a). Consistently, the protein half-life of TOP2β was extended by D4476 treatment but not by CX-4945 or GSK3i-IX treatment (Fig. 5b and Fig. S4A, B). Furthermore, we determined that the δ isoform, and ε isoform to a lesser extent, but not α isoform, of CK1 were involved in degrading TOP2β, as evidenced by the blockage of TOP2β degradation and the extension of its protein half-life upon CK1δ silencing via siRNA oligos (Fig. 5c, d, and Fig. S4C, D). Similar to ATM, we found an interaction between CK1δ and TOP2β, implying that CK1δ can directly bind to and phosphorylate TOP2β. Furthermore, this interaction was significantly increased in response to VM-26 treatment (Fig. 5e), suggesting that phosphorylation of TOP2β at Ser1134 induced by VM-26-activated ATM may promote CK1δ to phosphorylate TOP2β at Ser1130. Indeed, overexpression of CK1δ promoted the phosphorylation of TOP2β but had no effect on the S1130A mutant (Fig. 5f). Additionally, several studies showed that upon DNA damage, CK1δ is translocated from cytoplasm to nucleus^31,32^. Taken together, these results indicate that CK1 mediates TOP2β phosphorylation at Ser1130 to promote subsequent degradation in response to VM-26 treatment.

### Inactivation of SCF^β-TrCP^ ubiquitin ligase impairs DNA damage response by concealing DSBs induced by VM-26 treatment

**Fig. 6:**
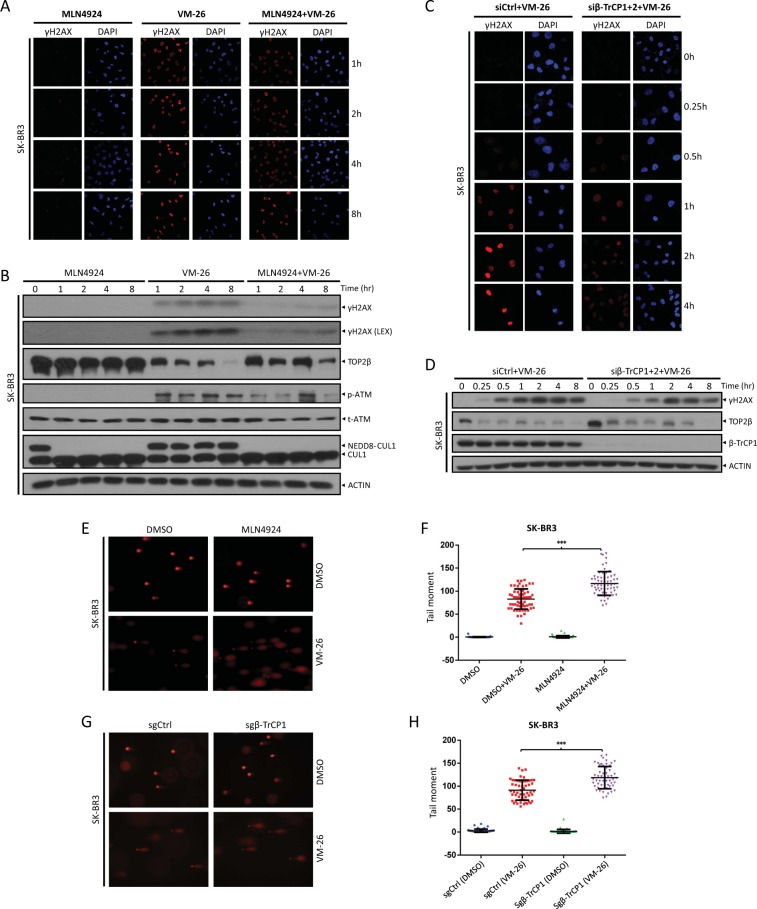
Inactivation of β-TrCP impairs DNA damage response by concealing DSBs upon VM-26 treatment. **a**, **b** MLN4924 attenuates DNA damage response upon VM-26 treatment. Cells treated with MLN4924 or VM-26 alone or in combination for the indicated time periods were harvested at the indicated time points for immunofluorescence (**a**) or IB (**b**) with the indicated Abs. LEX: longer exposure. **c**, **d** Silencing of β-TrCP attenuates DNA damage response upon VM-26 treatment. Cells transfected with siRNA targeting β-TrCP or scrambled control siRNA were treated with VM-26 for the indicated time periods and subjected to immunofluorescence (**c**) and IB (**d**) with the indicated Abs. (E-H) MLN4924 treatment or depletion of β-TrCP enhances VM-26-induced DSBs. SK-BR3 cells (**e**, **f**) were pretreated with MLN4924 (1 μM) for 1 h and then cotreated with VM-26 for an additional 1 h. SK-BR3 cells transfected with the indicated sgRNA (**g**, **h**) were treated with VM-26 for 1 h. Cells were then harvested for neutral comet assay. Representative images are shown (**e**, **g**). Comet tail moments were analyzed from at least 50 cells for each experimental condition, and the data are presented as the mean ± SEM from three independent experiments; ***p < 0.001 (**f**, **h**).

It has been reported that proteasome-mediated degradation of TOP2 cleavage complexes transforms TOP2 concealed DNA strand breaks into recognizable DSBs that trigger DNA damage signals, including ATM autophosphorylation at Ser1981 and the formation of γH2AX foci to recruit DNA repair machinery^7^. To determine whether SCF^β-TrCP^ ubiquitin ligase-mediated TOP2β degradation plays a physiological role in the VM-26-induced DNA damage response, we examined foci formation and the levels of γH2AX, a marker for DNA damage signaling^33^, in the presence of MLN4924 to block the activation of SCF^β-TrCP^ ubiquitin ligase. As expected, VM-26 significantly induced foci formation and increased the levels of γH2AX in SK-BR3 (Figs. 6a, b) and MDA-MB231 cells (Figs. S5A, B), indicating the induction of DNA damage signaling by VM-26. Notably, co-treatment of MLN4924, which inhibited TOP2β degradation (Fig. 6b and Fig. S5B), remarkably attenuated the foci formation and levels of γH2AX (Fig. 6a, b and Fig. S5A, B). Likewise, ATM autophosphorylation at Ser1981 triggered by VM-26 treatment was also reduced in the presence of MLN4924 (Fig. 6b and Fig. S5B). In addition, similar results were obtained when SCF^β-TrCP^ was specifically inactivated by siRNA-based knockdown (Fig. 6c, d and Fig. S5C, D). These results suggest that SCF^β-TrCP^-mediated TOP2β degradation plays vital roles in the transformation of TOP2-DNA covalent complexes into DSBs that are recognized by DNA damage signaling.

Next, to further support this notion, we used a neutral comet assay^34^ to measure the amount of DSBs. Notably, the comet tail moment identified by neutral comet assay reflected the total levels of TOP2 concealed DSBs and protein-free DSBs, as protease K was utilized in the lysis buffer to digest TOP2 in TOP2β-DNA covalent complexes to reveal DSBs concealed by TOP2β. As shown in Fig. 6e–h, VM-26 treatment, indeed, significantly increased the comet tail moment, indicating that DSBs were induced by VM-26, a finding that is consistent with the role of VM-26 in stabilizing TOP2β-DNA covalent complexes to subsequently induce DSBs. Co-treatment with MLN4924 (Fig. 6e, f) or β-TrCP1 depletion (Fig. 6g, h) caused a significant increase in DSBs compared with the treatment of VM-26 alone or with sgCtrl. This result implied that 1) blocking TOP2β degradation by inactivating SCF^β-TrCP^ stabilizes TOP2β-DNA covalent complexes and conceals DSBs, which impairs the recognition of damaged DNA to trigger DNA damage response and subsequent DNA repair, resulting in the increased DSBs observed in the neutral comet assay; 2) the attenuation of DNA damage response upon inactivation of SCF^β-TrCP^ by MLN4924 or β-TrCP depletion (Fig. 6a–d) was not caused by the reduction in DSBs. Taken together, our results clearly demonstrate that inactivation of SCF^β-TrCP^ ubiquitin ligase blocks TOP2β degradation induced by VM-26 treatment to conceal DSBs and subsequently attenuates DNA damage response.

### Depletion of β-TrCP or overexpression of β-TrCP-resistant TOP2β mutants sensitizes cancer cells to VM-26 treatment by promoting apoptosis

**Fig. 7:**
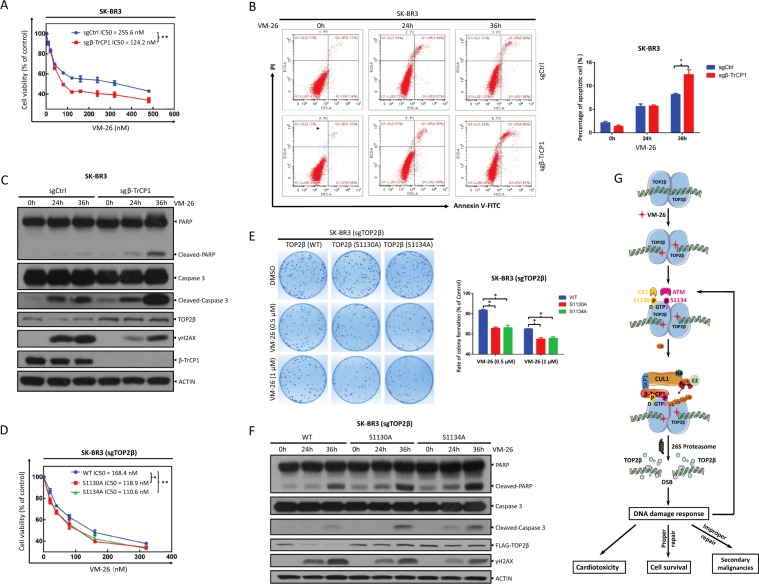
Depletion of β-TrCP1 and expression of β-TrCP-resistant TOP2β mutants sensitize cells to VM-26. **a** Depletion of β-TrCP1 sensitizes cells to VM-26. SK-BR3 cells plated in triplicate in 96-well plates were treated with various concentrations of VM-26 for 72 h and then subjected to an ATPlite assay. Data from three independent experiments are expressed as the mean ± SEM; **p < 0.01. **b**, **c** Depletion of β-TrCP1 promotes VM-26-induced cell apoptosis. SK-BR3 cells were treated with VM-26 for the indicated time periods and then subjected to FACS analysis to determine the apoptotic population (**b**, left, representative FACS profiles; right, the percentage of Annex V^+^ cells, mean ± SEM, *n* = 3, *p < 0.05) or IB with the indicated Abs (**c**). **d**–**f** Expression of β-TrCP-resistant TOP2β mutants sensitizes cells to VM-26. SK-BR3 cells depleted of endogenous TOP2β by sgRNA were transfected with TOP2β constructs and selected based on stable expression. Cells were then treated with VM-26 and then subjected to an ATPlite assay (**d**), clonogenic assay (**e**), and IB with the indicated Abs (**f**). For clonogenic assay, cells were pretreated with VM-26 (0.5 or 1 μM) for 1 h and cultured in fresh medium for 10 days. Data from three independent experiments are expressed as the mean ± SEM; *p < 0.05; **p < 0.01. **g** A model for SCF^β-TrCP^-mediated degradation of TOP2β promotes cancer cell survival in response to chemotherapeutic drugs targeting Topoisomerase II. See text for details.

Several lines of evidence have shown that chemotherapeutic drugs targeting topoisomerases induce DNA DSBs, leading to rapid TOP2β degradation and apoptotic cell death^7^. However, it is unknown whether TOP2β degradation induced by these chemotherapeutic drugs contributes to the killing of tumor cells. To this end, we determined the biological consequence of disrupting SCF^β-TrCP^-mediated TOP2β degradation triggered by VM-26 treatment. We first determined whether MLN4924, a small-molecule inhibitor of neddylation currently at the Phase I/II clinical trials against a number of human malignancies that inactivates all CRL ligases^20^, would enhance the killing of tumor cells by VM-26 treatment. We used an IC_20_ concentration of MLN4924 (Fig. S6A) in combination with various concentrations of VM-26 to determine the IC_50_ values of VM-26 with or without MLN4924. The ATPlite cell viability assay showed that MLN4924 caused a significant decrease in the IC_50_ values of VM-26 in SK-BR3 and H1299 cells from 107.1 nM to 49.9 nM and from 84.9 nM to 42.2 nM, respectively (Fig. S6B). More specifically, β-TrCP1 depletion caused a significant decrease in the IC_50_ values of VM-26 in SK-BR3 and H1299 cells from 255.6 nM to 124.2 nM and from 94.4 nM to 58.6 nM (Fig. 7a and Fig. S7A), respectively, indicating that cancer cells are more sensitive to VM-26 treatment after β-TrCP1 depletion. Mechanistically, the percentage of apoptotic cells, as reflected by Annexin V^+^ cells assessed by flow cytometry, was significantly increased when VM-26 treatment was combined with β-TrCP1 depletion (Fig. 7b and Fig. S7B). Likewise, VM-26 treatment markedly increased the cleavage of caspase-3 and PARP, two hallmarks of apoptosis, in β-TrCP1-depleted cells (Fig. 7c and Fig. S7C), suggesting that β-TrCP depletion sensitizes cancer cells to VM-26 by promoting apoptosis. Concomitantly, VM-26-induced TOP2β degradation and H2AX phosphorylation were also reduced in β-TrCP1-depleted cells, which suggested that blocking TOP2β degradation attenuates DNA damage response and DNA repair, ultimately leading to apoptosis induction (Fig. 7c and Fig. S7C).

To rule out the possible involvement of other β-TrCP substrates in VM-26 sensitization, we stably expressed wild-type (WT) TOP2β or TOP2β (S1130A) and TOP2β (S1134A), two TOP2β mutants resistant to SCF^β-TrCP^-mediated degradation, in cells with depletion of endogenous TOP2β by the CRISPR-Cas9 approach. We found that ectopic expression of β-TrCP-resistant TOP2β mutants sensitized cells to VM-26 treatment, as evidenced by (1) the decrease in IC_50_ values (Fig. 7d and Fig. S7D) and clonogenic cell survival (Fig. 7e and Fig. S7E), and (2) the increase in apoptosis (Fig. 7f and Fig. S7F) in cells expressing TOP2β-S1130A or TOP2β-S1134A compared to those in cells expressing TOP2β-WT. Taken together, our results demonstrate that blocking β-TrCP-mediated TOP2β degradation attenuates DNA damage response to impair DNA repair, leading to enhanced apoptosis and greater sensitivity to VM-26 treatment.

## Discussion

In this study, we identified and characterized TOP2β, a type II topoisomerase, as a physiological substrate of SCF^β-TrCP^ E3 ubiquitin ligase. Our conclusion is supported by the following lines of evidence: (1) inactivating SCF E3 ubiquitin ligases by MLN4924 blocks TOP2β degradation induced by VM-26 treatment, extends the TOP2β half-life, and reduces TOP2β polyubiquitination; (2) TOP2β binding to β-TrCP, which is dependent on an evolutionarily conserved β-TrCP binding motif on TOP2β, is enhanced significantly upon VM-26 treatment, leading to TOP2β polyubiquitination; (3) inactivating SCF^β-TrCP^ E3 ubiquitin ligase by silencing CUL1 or β-TrCP blocks TOP2β degradation and extends its protein half-life; (4) ATM and CK1 phosphorylate TOP2β at Ser1134 and Ser1130 within the β-TrCP binding motif, respectively; and (5) inactivating ATM or CK1 by siRNA knockdown, gene knockout and small-molecule inhibitor blocks TOP2β degradation and extends its protein half-life. Biologically, we demonstrated that SCF^β-TrCP^-mediated TOP2β degradation promotes cancer cell survival by promoting DNA damage signals that facilitate DNA repair, whereas blockage of this degradation promotes cell killing via enhancing apoptosis (Fig. 7g).

A previous study showed that proteasomal inhibitors significantly enhance the growth inhibition of drugs targeting DNA topoisomerase II, indicating that blocking TOP2 degradation is an attractive strategy to sensitize cancer cells to TOP2-targeting chemotherapeutic drugs (TOP2 poisons)^35^. Although TOP2 poisons induce the degradation of both TOP2α and TOP2β, the rate and extent of degradation are much greater for TOP2β than for TOP2α (Fig. 2c and Fig. S1B)^13^, a finding that provides novel insight that may explain why the cytotoxic effects of most TOP2 poisons in tumor cells appear to be mainly TOP2α-dependent. Therefore, specifically blocking the degradation of TOP2β would be a better choice for effectively enhancing the anticancer efficacy of TOP2 poisons. However, the proteasomal inhibitors used in a previous study are associated with high normal cell toxicity, as a result of the global inhibition of protein degradation through the 26 S proteasome^35^. In this study, we revealed the precise mechanism by which TOP2 poisons induce TOP2β degradation and demonstrated that specifically blocking TOP2β degradation remarkably sensitizes cancer cells to VM-26 treatment (Fig. 7). Thus, our study suggests a novel strategic drug combination of TOP2 poisons with an inhibitor targeting TOP2β degradation for maximal killing efficiency of tumor cells.

Chemotherapeutic drugs targeting topoisomerases are widely used in anticancer treatment in the clinic^6^. For example, etoposide (VP-16) is approved to treat lymphomas, nonlymphocytic leukemia, and solid tumors^8^; teniposide (VM-26) is used in the treatment of acute lymphoblastic leukemia (ALL) in children, lymphoma, and brain cancer^6^; and doxorubicin acts as a potent anticancer drug and is approved for the treatment of many human cancers, including solid tumors (such as breast, lung, ovarian, bladder, liver, and thyroid), lymphomas, and ALL^9^. Unfortunately, these drugs are associated at a high incidence with development of secondary malignancies. For example, a specific type of leukemia was developed in patients treated with etoposide or doxorubicin, likely due to induced chromosomal translocations^36^. Recent studies have shown that TOP2β-DNA cleavage complexes have a preferential role in the generation of leukemia chromosomal break points^14,36^. Thus, at least in theory, small inhibitors targeting TOP2β degradation will reduce the leukemogenic potential of TOP2 poisons. Therefore, the mechanism underlying TOP2β degradation elucidated in our study may help to uncover a new way to decrease the incidence of secondary malignancies induced by the treatment of TOP2 poisons, while enhancing their anticancer efficacy.

Few recent studies have shown that TOP2β-mediated DNA DSBs that subsequently induce DNA damage signals contribute to the cardiotoxicity induced by doxorubicin^37,38^. Proteasomal degradation of TOP2β exposes the TOP2β-concealed DNA DSBs that results in genomic instability, which could play an important role in the development of this side effect. Thus, proteasome inhibitors that block TOP2β degradation to reduce DNA damage signals or a knockout of TOP2β would likely prevent doxorubicin-induced cardiotoxicity. Indeed, mice carrying cardiomyocyte-specific deletion of Top2β were found to be significantly protected from the development of doxorubicin-induced progressive heart failure^38^, indicating that TOP2β is a promising molecular target for preventing cardiotoxicity induced by doxorubicin or other TOP2 poisons^39–41^. Our findings reported in this study, therefore, have a translational implication by providing a more precise strategy to prevent doxorubicin cardiotoxicity through blockage of TOP2β degradation.

In summary, our study established a feed-forward loop by which TOP2 poisons trigger DNA damage signal for instant TOP2β degradation, which then fully expose damaged DNA to triggers more DNA damage response and repair. Specifically, upon exposure to TOP2 poisons, TOP2β-DNA complexes were stabilized which disrupted TOP2-mediated religation of the broken strands, leading to ATM activation. Activated ATM then phosphorylated TOP2β at Ser1134 to trigger CK1 phosphorylation of TOP2β at Ser1130 on the β-TrCP degron motif, which is then recognized by β-TrCP for binding and subsequent ubiquitination by SCF E3 ligase for proteasomal degradation. The degradation of TOP2β exposes TOP2β-concealed DNA DSBs to enhance DNA damage signals that further activates ATM and initiates DNA repair for cell survival. Thus, the small molecules that inhibit TOP2β degradation would disrupt this feed-forward loop to reduce DNA damage response and repair for enhanced cancer cell killing. At the same time, termination of this feed-forward loop may also reduce cardiotoxicity and secondary malignancies induced by TOP2 poisons (Fig. 7g).

## Materials and methods

### Cell lines and chemicals

All cell lines, SK-BR3, MDA-MB231, MCF7, HEK293, and H1299 were obtained from American Type Culture Collection (ATCC), and maintained in Dulbecco’s modified Eagle’s medium, supplemented with 10% (v/v) fetal bovine serum (FBS) and 1% penicillin/streptomycin at 37 °C in a humidified incubator with 5% CO_2_. The following chemicals were obtained from commercial sources: MLN4924 (Apexbio), MG132 (Cayman), CHX (Sigma), VM-26 (Sigma), KU60019 (MCE), AZD6738 (MCE), LTURM34 (MCE), D4476 (Selleck), GSK3i-IX (Selleck), and CX-4945 (Selleck).

### Cell transfection and siRNA silencing

For cell transfection, sub-confluent cells were transfected with the corresponding plasmids or siRNA oligos using Lipofectamine 3000 (Invitrogen), according to the manufacturer’s instructions. The sequences of siRNA oligos are as follows: siCtrl: 5′-ATT GTA TGC GAT CGC AGA C-3′; siCUL1: 5′-GGT CGC TTC ATA AAC AAC A-3′; siβTrCP1 + 2: 5′-AAG TGG AAT TTG TGG AAC ATC-3′; siATM: 5′-AAC ATA CTA CTC AAA GAC ATT-3′; siCK1α: 5′-GGC TAA AGG CTG CAA CAA A-3′; siCK1δ: 5′-CCA AGA GAC AGA AAT ACG AAA-3′; siCK1ε: 5′-GCG ACT ACA ACG TGA TGG T-3′.

### CRISPR/Cas9-mediated knockout

Human breast cancer SK-BR3 and MDA-MB231 cells and human lung cancer H1299 cells with β-TrCP1 or TOP2β knockout were generated by the CRISPR-Cas9 system. Briefly, single-guide RNA (sgRNA) against β-TrCP1 or TOP2β was subcloned into the plasmid pSpCas9(BB)-2A-Puro (PX459). Cells were transfected with the construct and selected with puromycin for 3 days, and single clones were selected under a microscope.

### Immunoblotting and immunoprecipitation

For immunoblotting (IB), cells were lysed in buffer supplemented with phosphatase inhibitors and protease inhibitors, followed by ultrasound. The lysates were then separated on SDS-PAGE and immunoblotted with the indicated antibodies (Abs). For immunoprecipitation (IP), whole-cell extract was incubated with the corresponding antibody in a rotating incubator for 3–5 h at 4 °C, to which Protein A/G Sepharose beads were added, and the incubation continued for an additional 2–4 h. The immunoprecipitates were then washed four times with lysis buffer and subjected to IB with the indicated Abs, as previously described^42^.

The following antibodies were used: TOP2β (611493, BD Biosciences, 1:1000), TOP2α (12286, Cell Signaling Technology, 1:1000), CUL1 (sc-11384, Santa Cruz, 1:1000), ACTIN (A5441, Sigma, 1:10000), NEDD8 (ab81264, Abcam, 1:4000), β-TrCP1 (4394, Cell Signaling Technology, 1:1000), FLAG (F1804, Sigma, 1:2000), p-AKT (4060, Cell Signaling Technology, 1:2000), AKT (4691, Cell Signaling Technology, 1:2000), p-ATR (2853, Cell Signaling Technology, 1:1000), p-ATM (200-301-400S, Rockland, 1:1000), ATM (2873, Cell Signaling Technology, 1:1000), β-Catenin (9582, Cell Signaling Technology, 1:1000), CK1δ (12417, Cell Signaling Technology, 1:500), γH2AX (05-636, Millipore, 1:10000), PARP (9542, Cell Signaling Technology, 1:1000), Caspase 3 (9665, Cell Signaling Technology, 1:1000), p-CHK1 (2348, Cell Signaling Technology, 1:2000), CHK1 (sc-8408, Santa Cruz, 1:1000), and p-S/T (9631, Cell Signaling Technology, 1:1000).

### Flow cytometry

Cells were treated with VM-26 for various time periods and then stained with an annexin V-FITC/propidium iodide (PI) apoptosis detection kit (Beyotime Biotechnology, Shanghai, China), according to the manufacturer’s instructions, and then counted by flow cytometry, as described previously^43^.

### In vivo ubiquitination assay

HEK293 cells were transfected with various plasmids with PolyJet (SignaGen Laboratories), according to the manufacturer’s instructions. After 48 h, cells were treated with VM-26 or MLN4924, along with MG132, as described. Cells were then lysed in buffer containing phosphatase inhibitors and protease inhibitors, pulled down with HA beads and subjected to direct IB using anti-TOP2β or anti-FLAG antibody.

### ATPlite assay

A total of 2000 cells were seeded in triplicate in 96-well plates and treated with VM-26 at various concentrations for 72 h and then assayed for viability with ATPlite, according to the manufacturer’s instructions for use of the ATPlite 1 step Luminescence Assay System (PerkinElmer), as previously described^44^. The results obtained from three independent experiments were plotted.

### Neutral comet assay

Neutral comet assays were performed as previously described^34^. In brief, cells were cultured in 60-mm dishes and treated with 2 mM thymidine. Twenty-four hours later, the cells were washed once with phosphate-buffered saline (PBS) and incubated for additional 1 h with VM-26 or MLN4924 alone or in combination. Cells were then harvested and coated onto the slide. For cellular lysis, slides were immersed in neutral N1 lysis solution (0.5 mg/ml proteinase K, 0.5 M EDTA, 2% sarkosyl, pH 8.0) overnight at 37 °C. Then, the slides were stained with 10 μg/ml propidium iodide (PI) for 20 min, and the cells were subjected to electrophoresis at 15 V for 25 min (0.6 V/cm) and viewed using a fluorescence microscope. The comet tail moment was analyzed by the CometScore software.

### Immunofluorescence

Following treatment, cells were fixed and stained with anti-γH2AX antibody and DAPI as previously described^45^. Cells were then photographed with a Nikon A1 Ti confocal microscope (Nikon, Japan) using a ×60 oil objective lens.

### Statistical analysis

The data from three independent experiments are presented as the mean ± SEM. A two-tailed Student’s *t* test for statistical analysis was performed with the Prism software (GraphPad). Statistical significance was determined for differences with *p* < 0.05.

## Supplementary information

Supplementary Figure Legends

Figure S1

Figure S2

Figure S3

Figure S4

Figure S5

Figure S6

Figure S7
