# Supplementary material for: SCFβ-TrCP-mediated degradation of TOP2β promotes cancer cell survival in response to chemotherapeutic drugs targeting topoisomerase II
Source: Oncogenesis. 2020 Feb 3;9(2):8. doi: 10.1038/s41389-020-0196-1 (PMC6997367; doi:10.1038/s41389-020-0196-1)
Supplement: Supplementary file 1 — Supplementary Figure Legends [file 41389_2020_196_MOESM1_ESM.pdf]

Supplemental information

Supplemental figure legends

Figure S1. MG132 and MLN4924 inhibit TOP2β degradation. (A) MG132 blocks TOP2β degradation. SK-BR3 and MDA-MB231 cells were treated with MG132 for the indicated time periods, and then, IB was undertaken with the indicated Abs. (B) Instant TOP2β degradation by multiple chemotherapeutic drugs targeting Topoisomerase II. Cells were treated with VP-16 (200 μM), VM-26 (100 μM), m-AMSA (100 μM), doxorubicin (DOX, 100 μM), and camptothecin (CPT, 25 μM) for 2 hrs, and then subjected to IB analysis with the indicated Abs. (C) MLN4924 inhibits TOP2β degradation induced by VM-26. SK-BR3 pretreated with MG132 or MLN4924 for 2 hrs were treated with CHX and VM-26. Cells were then harvested at indicated time points for IB with indicated Abs (top). Densitometry quantification was performed with ImageJ, and the decay curves are shown (bottom).

Figure S2. Inactivation of $SCF^{\beta\text{-}TrCP}$ ubiquitin ligase extends the protein half-life of TOP2β upon VM-26 treatment. (A) CUL1 silencing extends the protein half-life of TOP2β upon VM-26 treatment. SK-BR3 and MDA-MB231 cells transfected with siRNA targeting CUL1 or scrambled control siRNA were treated with VM-26 and CHX, followed by IB with indicated Abs. (B) Depletion of β-TrCP1 extends the protein half-life of TOP2β. Cells were transfected with sgRNA based on CRISPR-Cas9 mediated knockout. Single cell clones were sorted and then treated with CHX and VM-26 for various time periods, followed by IB with indicated Abs (left). Densitometry quantification was performed with ImageJ, and the decay curves are shown (right).

Figure S3. Inhibition of ATR and DNA-PK has no effects on VM-26 induced TOP2β degradation. (A) VM-26 treatment activates ATM. SK-BR3 and MDA-MB231 cells were treated with VM-26 (100 μM) for indicated time periods, followed by IB with indicated Abs. (B, C) Inhibition of ATR and DNA-PK has no effects on VM-26 induced TOP2β degradation. SK-BR3 and MDA-MB231 cells pretreated with AZD6738 (1 μM) (B) or LTURM34 (5 μM) (C) for 1 hr were treated with CHX and VM-26 for indicated time periods, followed by IB with indicated Abs (left).

Densitometry quantification was performed with ImageJ, and the decay curves are shown (right).

Figure S4. Inhibition of GSK3 and CK2 or silencing of CK1α and CK1ε has no effects on VM-26 induced TOP2β degradation. SK-BR3 and MDA-MB231 cells pretreated with CX-4945 (10 μM) (A) or GSK3i-IX (100 μM) (B) for 4 hrs were treated with CHX and VM-26 for various time periods, followed by IB with indicated Abs (left). Densitometry quantification was performed with ImageJ, and the decay curves are shown (right). (C, D) Silencing of CK1α and CK1ε isoforms has minimal effects on TOP2β turnover. SK-BR3 and MDA-MB231 cells transfected with siRNA oligos targeting CK1α (C) and CK1ε (D) or scrambled control siRNA were treated with CHX and VM-26 for various time periods, followed by IB with indicated Abs (left). Densitometry quantification was performed with ImageJ, and the decay curves are shown (right).

Figure S5. Inactivation of $SCF^{\beta\text{-}TrCP}$ ubiquitin ligase impairs VM-26-triggered DNA damage response. (A and B) MLN4924 attenuates DNA damage response upon VM-26 treatment. MDA-MB231 cells were treated with MLN4924 or VM-26 alone, or in combination for indicated time periods, followed by immunofluorescence (A) and IB (B) with indicated Abs. (C and D) Silencing of β-TrCP attenuates DNA damage response upon VM-26 treatment. MDA-MB231 cells transfected with siRNA oligos targeting β-TrCP or scrambled control siRNA were treated with VM-26 for indicated time periods, followed by immunofluorescence (C) and IB (D) with indicated Abs.

Figure S6. MLN4924 sensitizes cells to VM-26. (A) The IC_{20} concentrations of MLN4924 in SK-BR3 and H1299 cells. Cells plated in triplicate in 96-well plates were treated with various concentrations of MLN4924 for 72 hrs and then subjected to an ATPlite assay. (B) MLN4924 sensitizes cells to VM-26. SK-BR3 and H1299 cells were treated with various concentrations of VM-26 alone or in combination of an IC_{20} concentration of MLN4924 (SK-BR3 80 nM; H1299 10 nM) and then subjected to an ATPlite assay. Data from three independent experiments are presented as the mean ± SEM; ***$p < 0.001$.

Figure S7. Depletion of β-TrCP1 and expression of β-TrCP-resistant TOP2β mutants sensitize cells to VM-26. (A) Depletion of β-TrCP1 sensitizes cells to VM-26. H1299 cells plated in triplicate in 96-well plates were treated with various concentrations of VM-26 for 72 hrs and then subjected to an ATPlite assay. Data from three independent experiments are expressed as the mean ± SEM; $^{**}p < 0.01$. (B, C) Depletion of β-TrCP1 promotes VM-26-induced cell apoptosis. H1299 cells were treated with VM-26 for the indicated time periods and then subjected to FACS analysis to determine the apoptotic population (B, left, representative FACS profiles; right, the percentage of Annex V^+ cells, mean ± SEM, n=3, $^{*}p < 0.05$) or IB with the indicated Abs (C). (D-F) Expression of β-TrCP-resistant TOP2β mutants sensitizes cells to VM-26. H1299 cells depleted of endogenous TOP2β by sgRNA were transfected with TOP2β constructs and selected based on stable expression. Cells were then treated with VM-26 and then subjected to an ATPlite assay (D), clonogenic assay (E), and IB with the indicated Abs (F). For clonogenic assay, cells were pretreated with VM-26 (0.5 or 1 μM) for 1 hr and cultured in fresh medium for 8 days. Data from three independent experiments are expressed as the mean ± SEM; $^{*}p < 0.05$; $^{**}p < 0.01$.
