# Supplementary figures and images for: SCFβ-TrCP-mediated degradation of TOP2β promotes cancer cell survival in response to chemotherapeutic drugs targeting topoisomerase II
Source: Oncogenesis. 2020 Feb 3;9(2):8. doi: 10.1038/s41389-020-0196-1 (PMC6997367; doi:10.1038/s41389-020-0196-1)

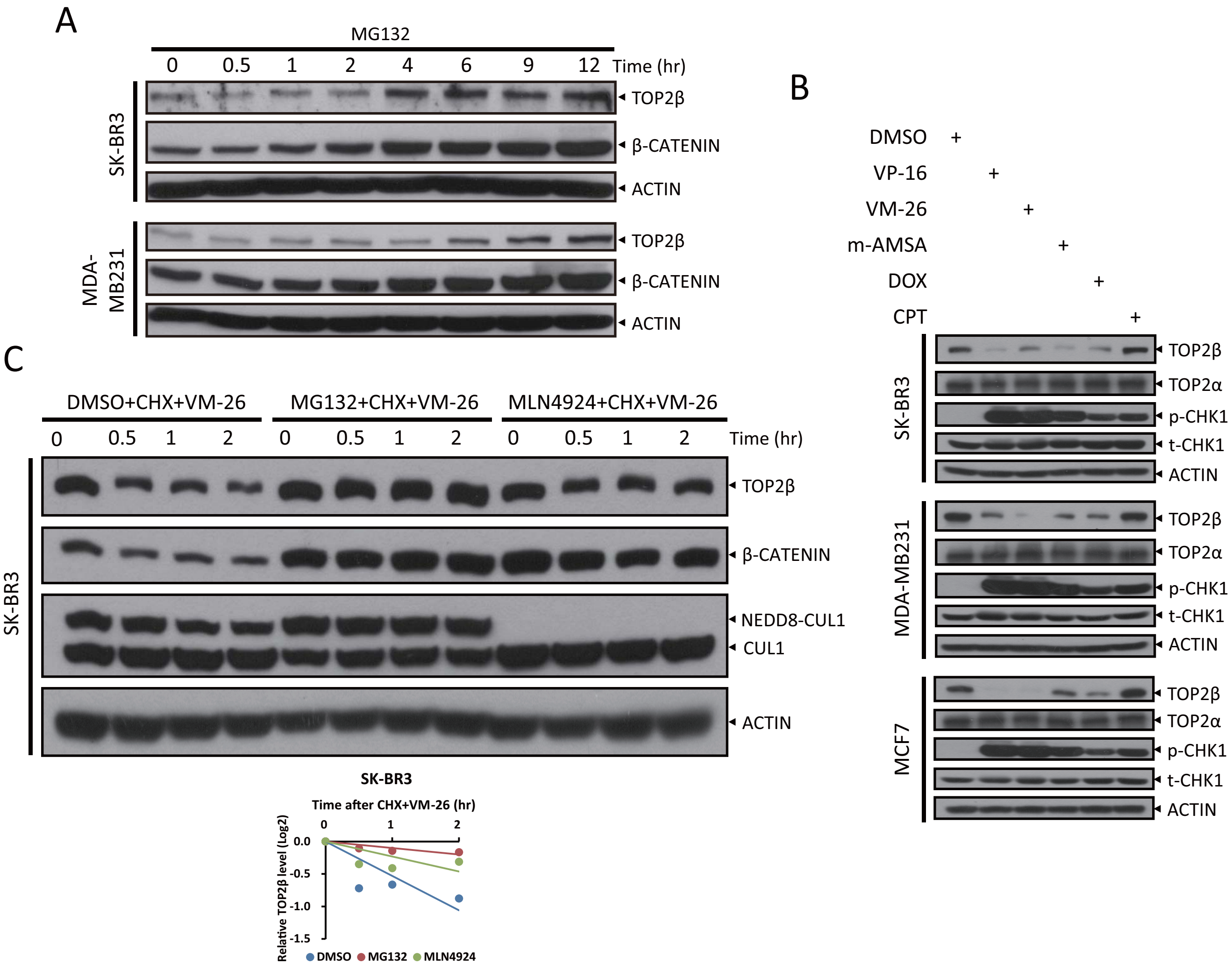

Supplement: Supplementary file 2 — Figure S1 [file 41389_2020_196_MOESM2_ESM.pdf]

A

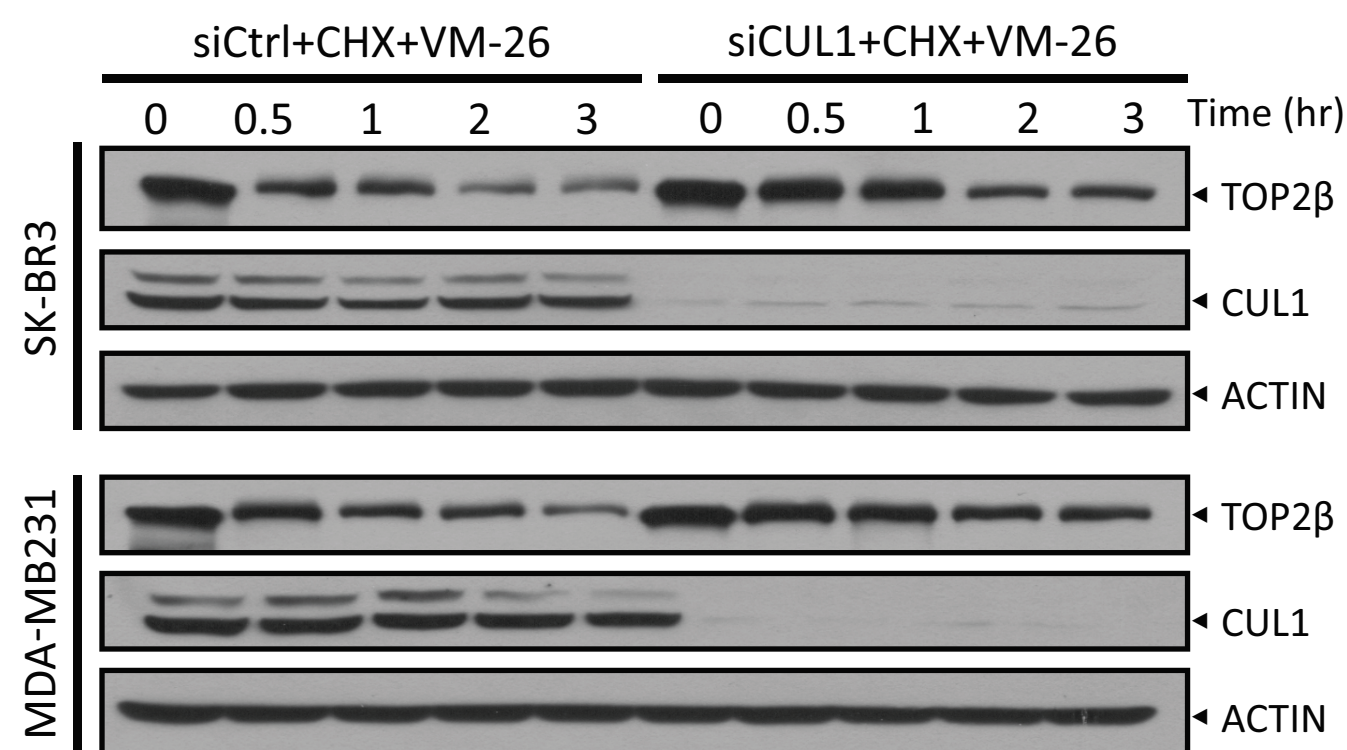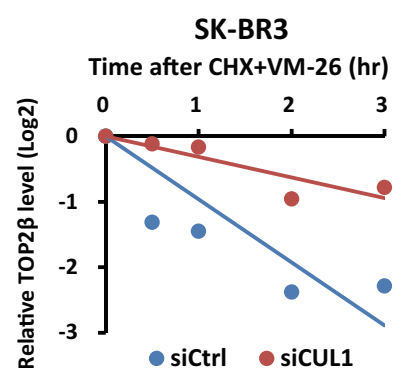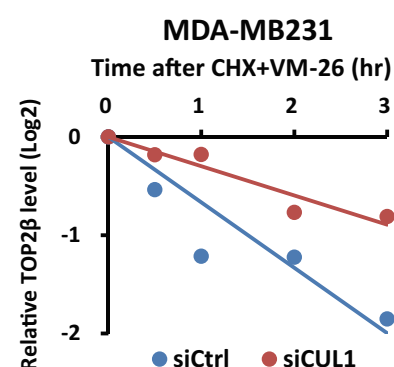

B

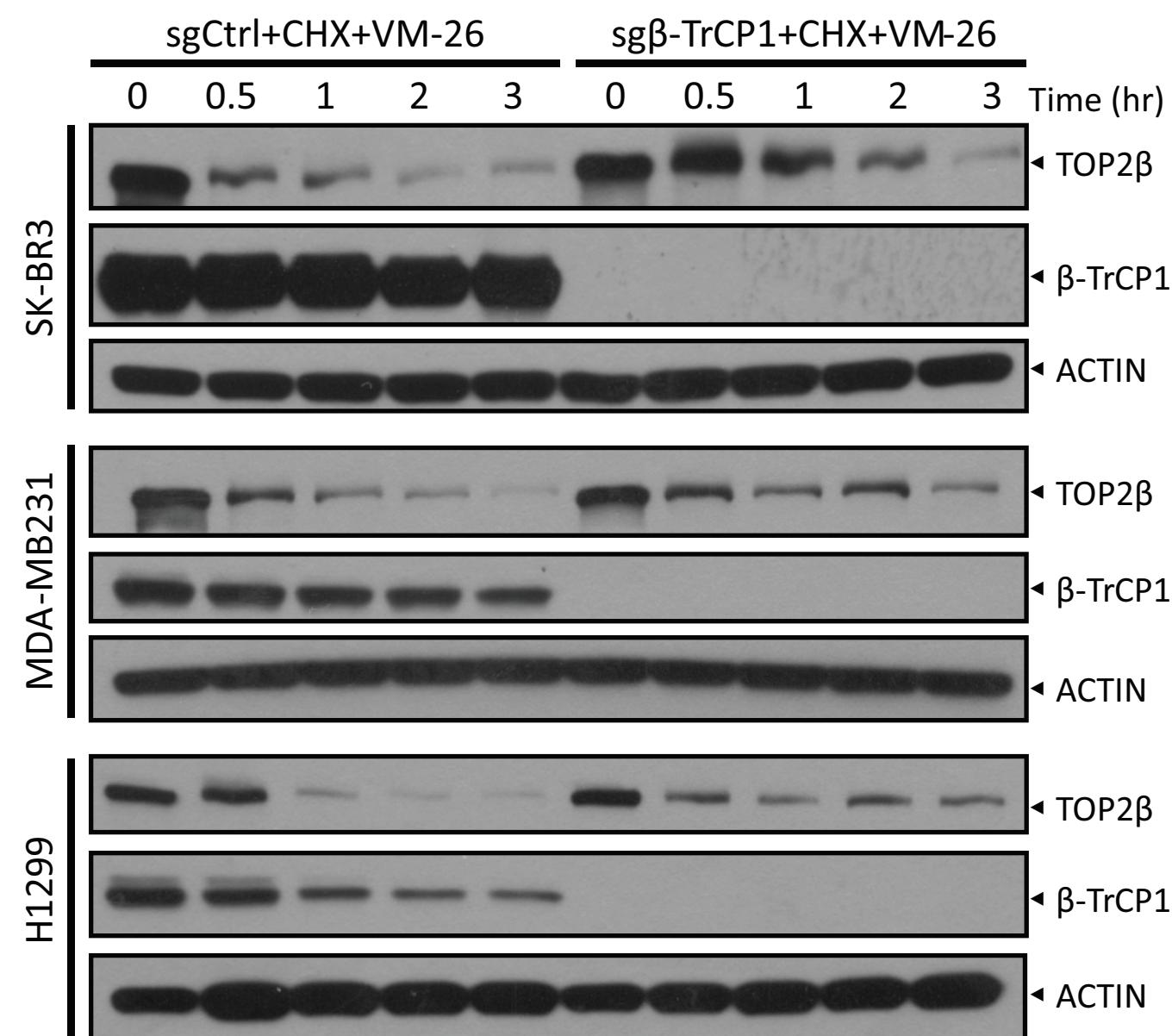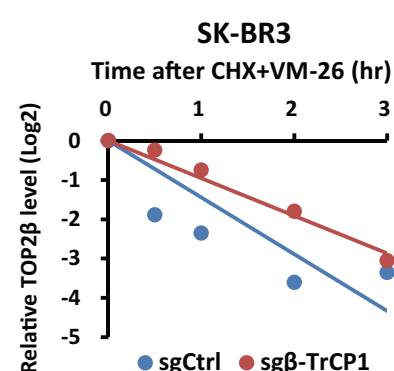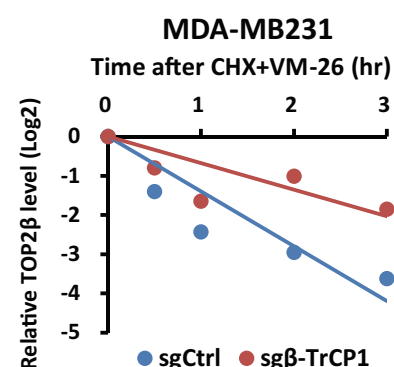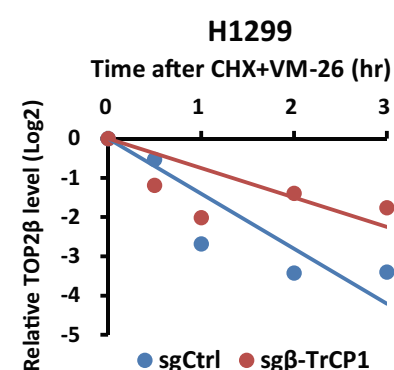

Supplement: Supplementary file 3 — Figure S2 [file 41389_2020_196_MOESM3_ESM.pdf]

A

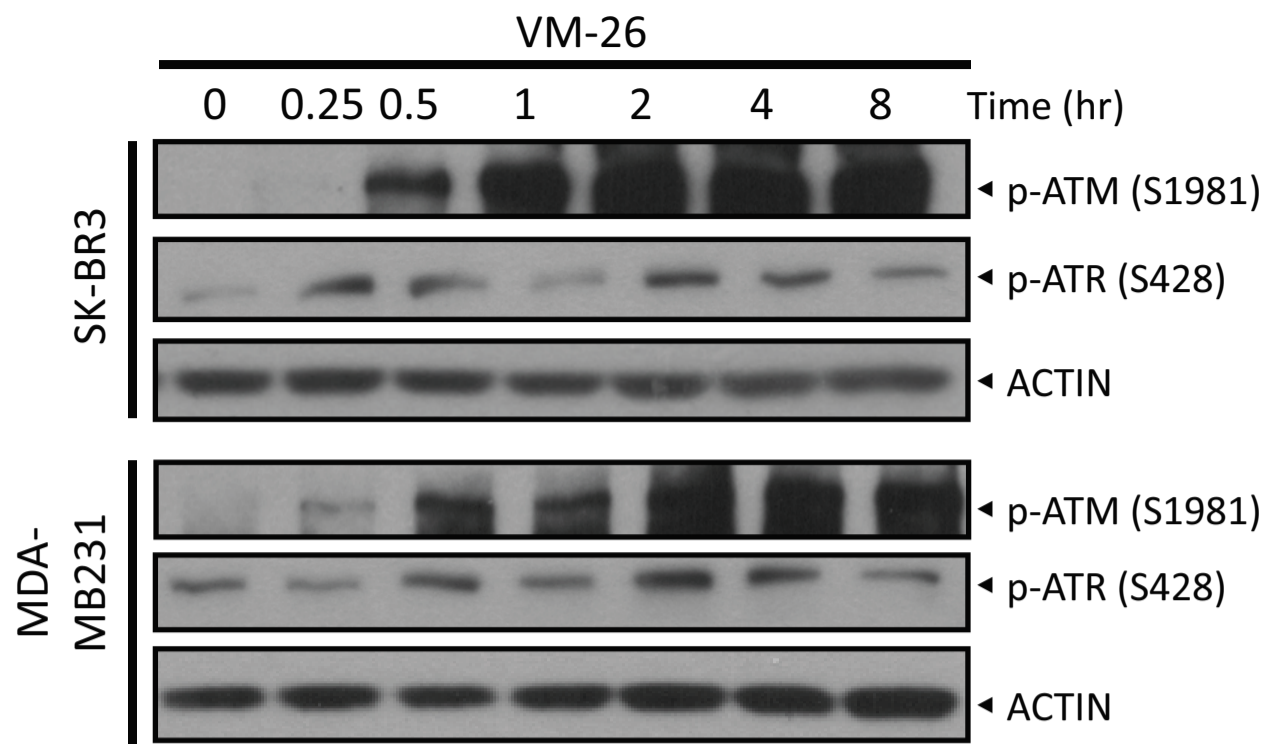

B

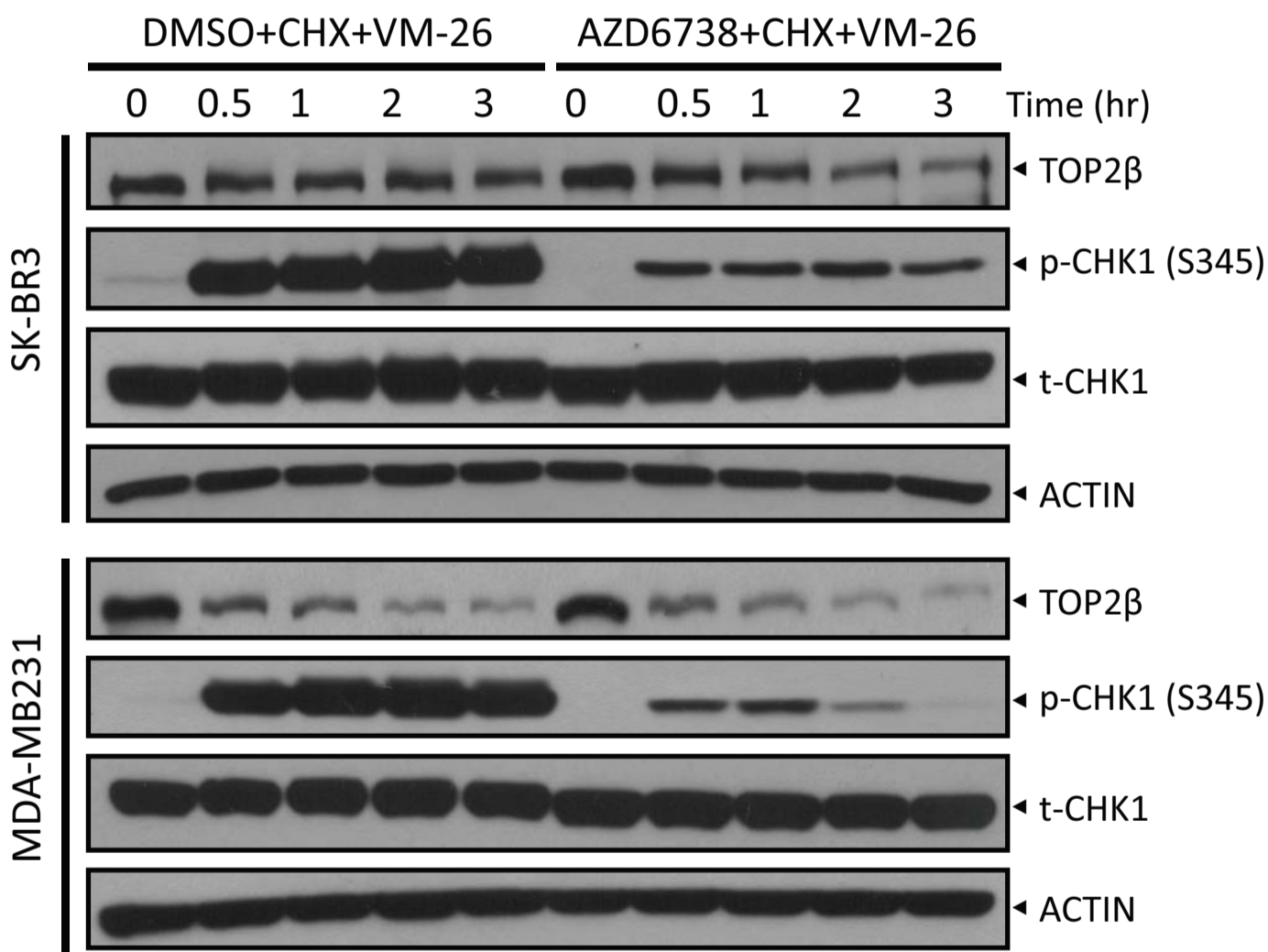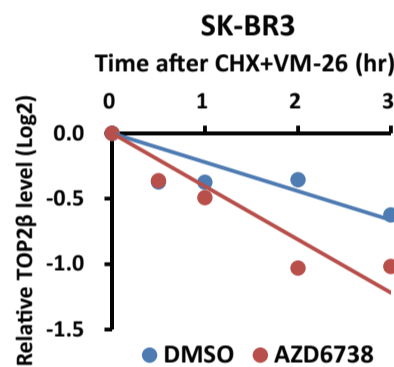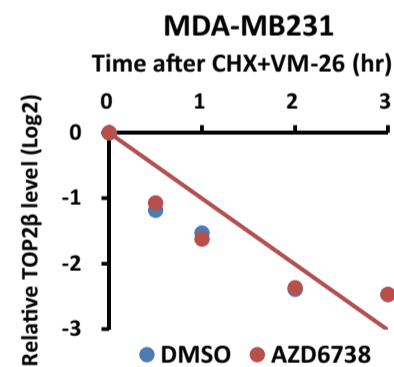

C

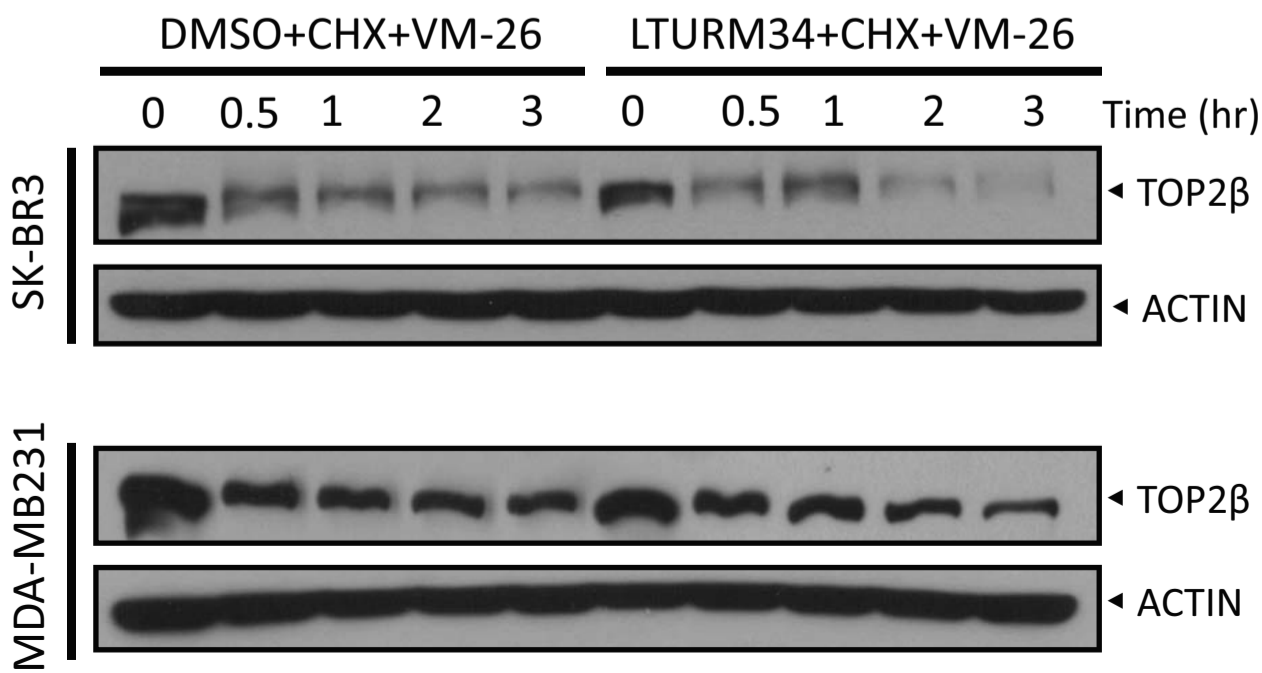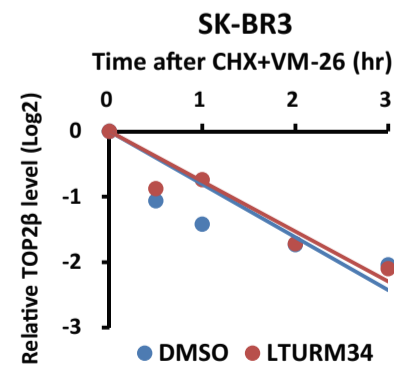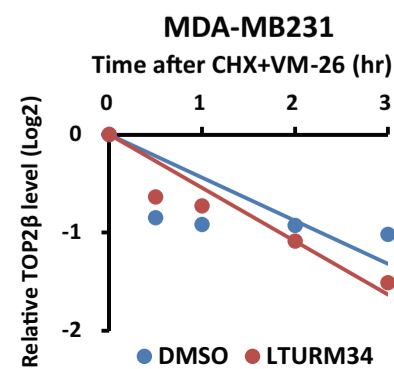

Supplement: Supplementary file 4 — Figure S3 [file 41389_2020_196_MOESM4_ESM.pdf]

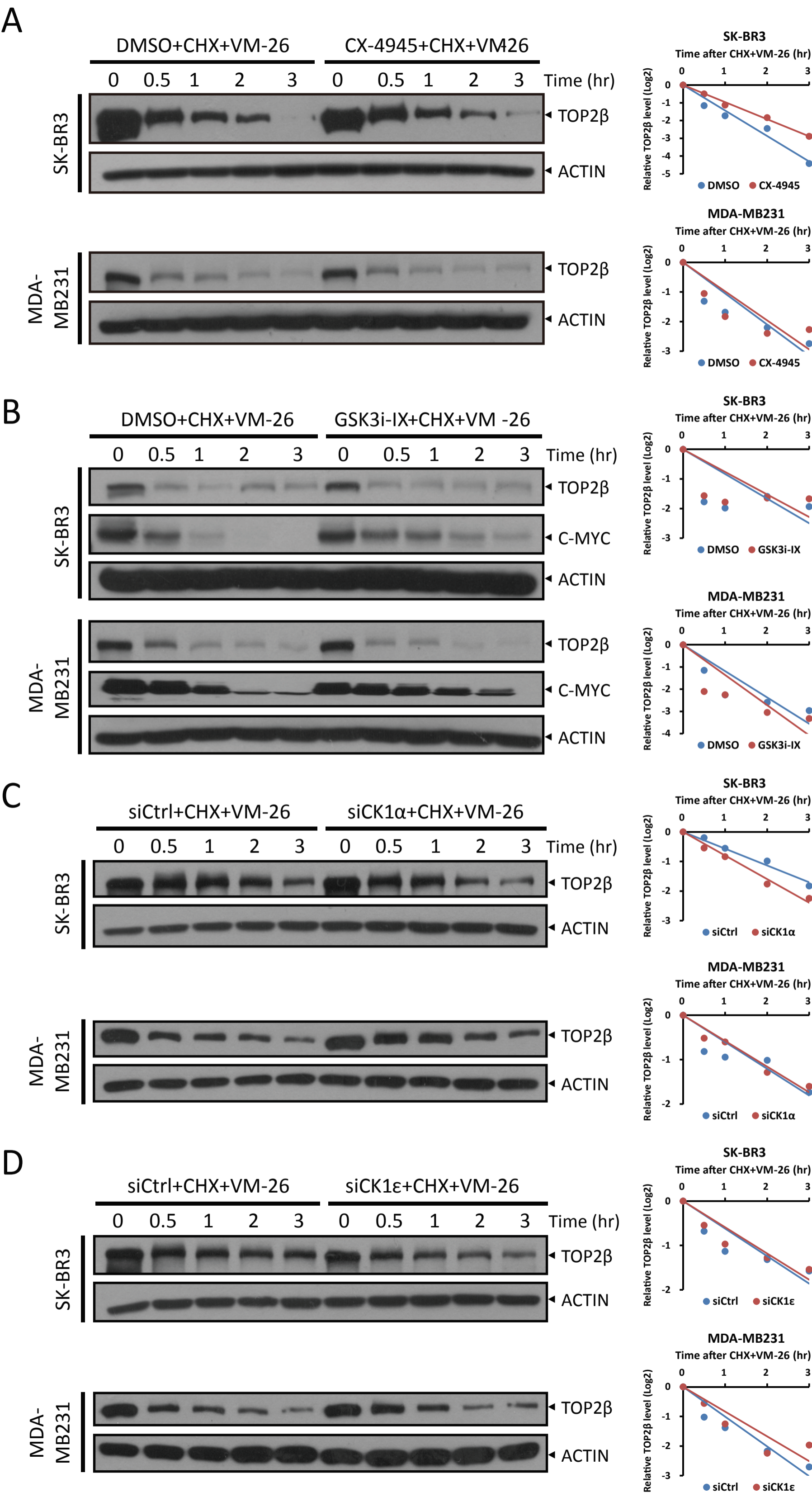

Supplement: Supplementary file 5 — Figure S4 [file 41389_2020_196_MOESM5_ESM.pdf]

A

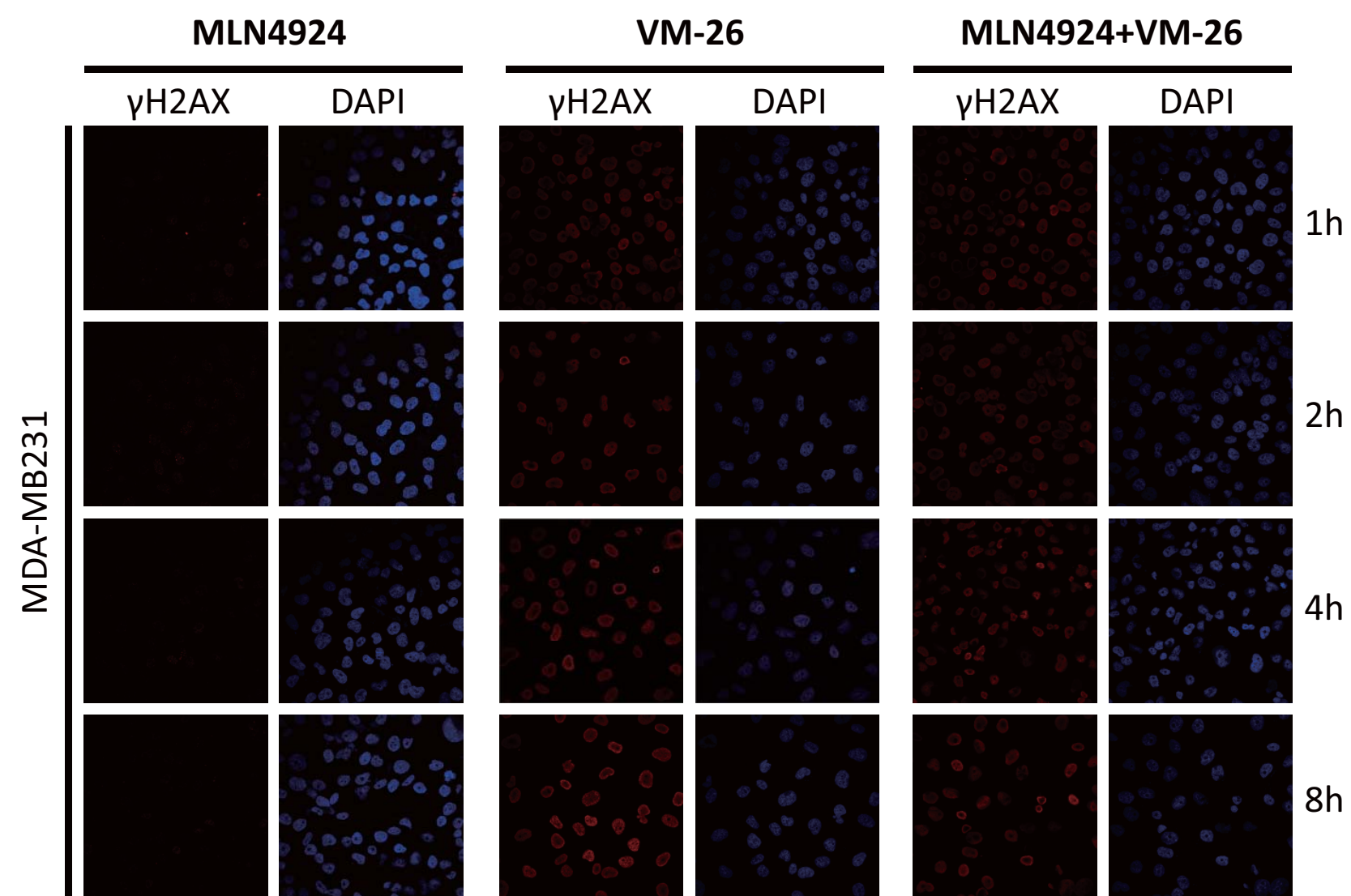

B

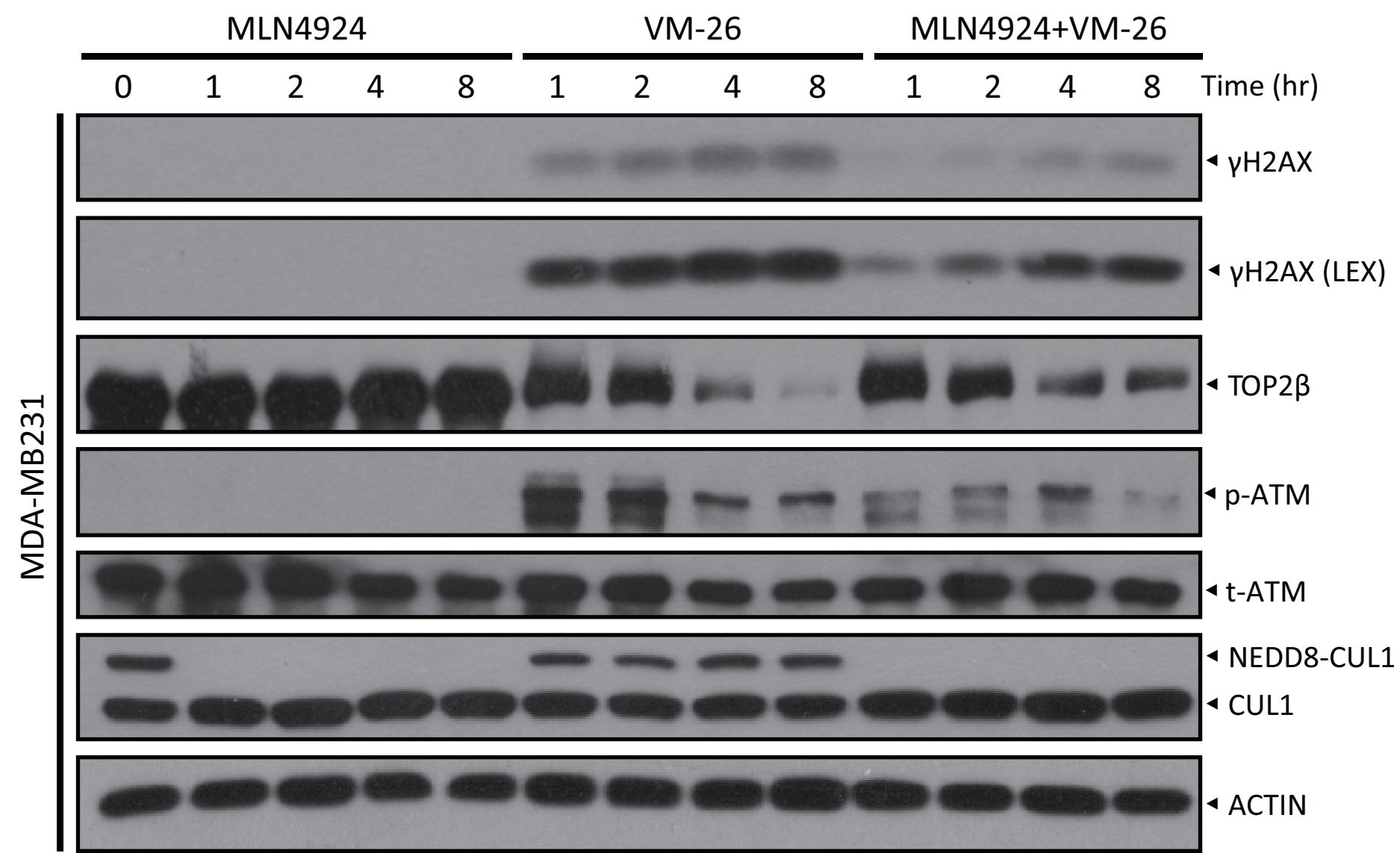

C

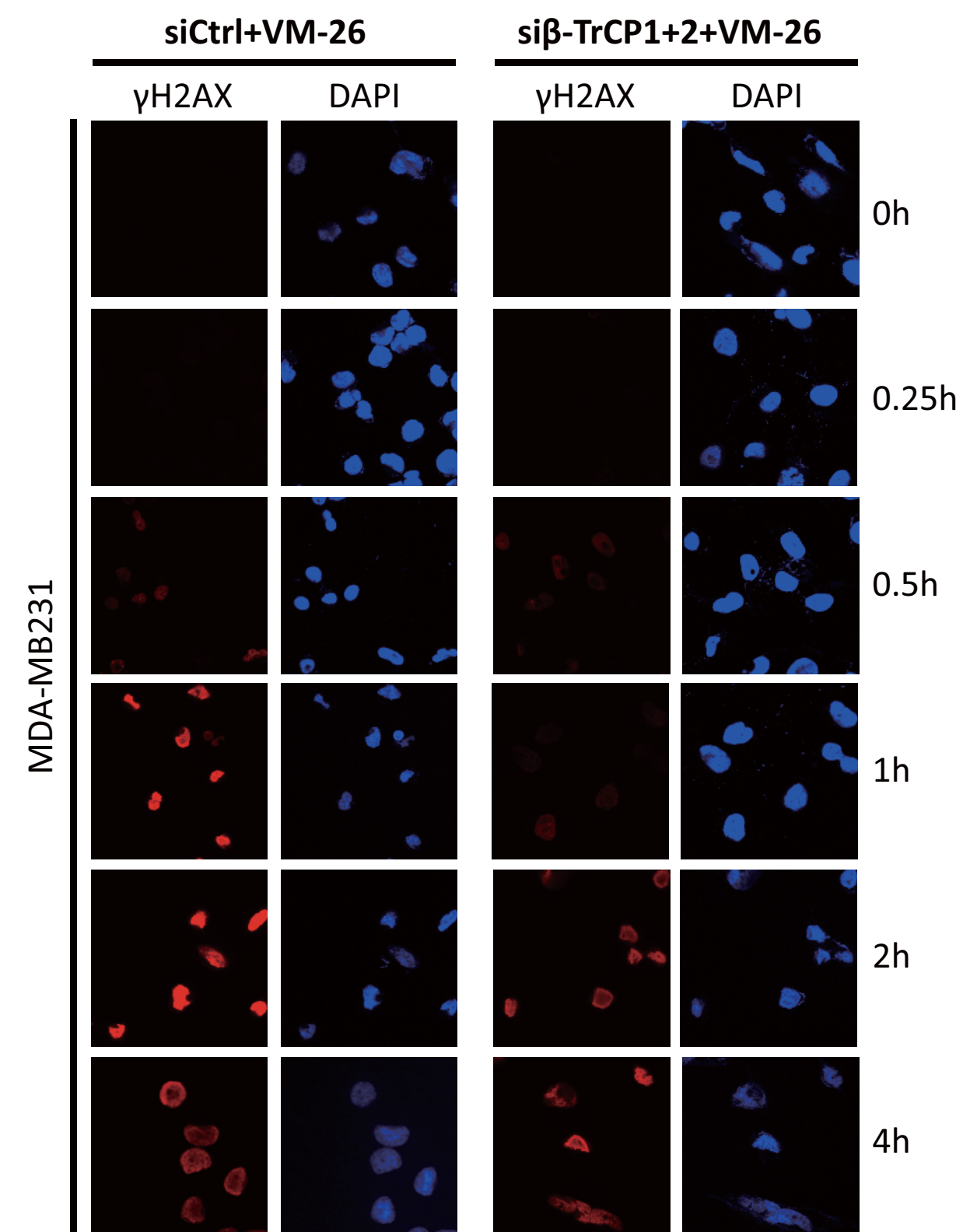

D

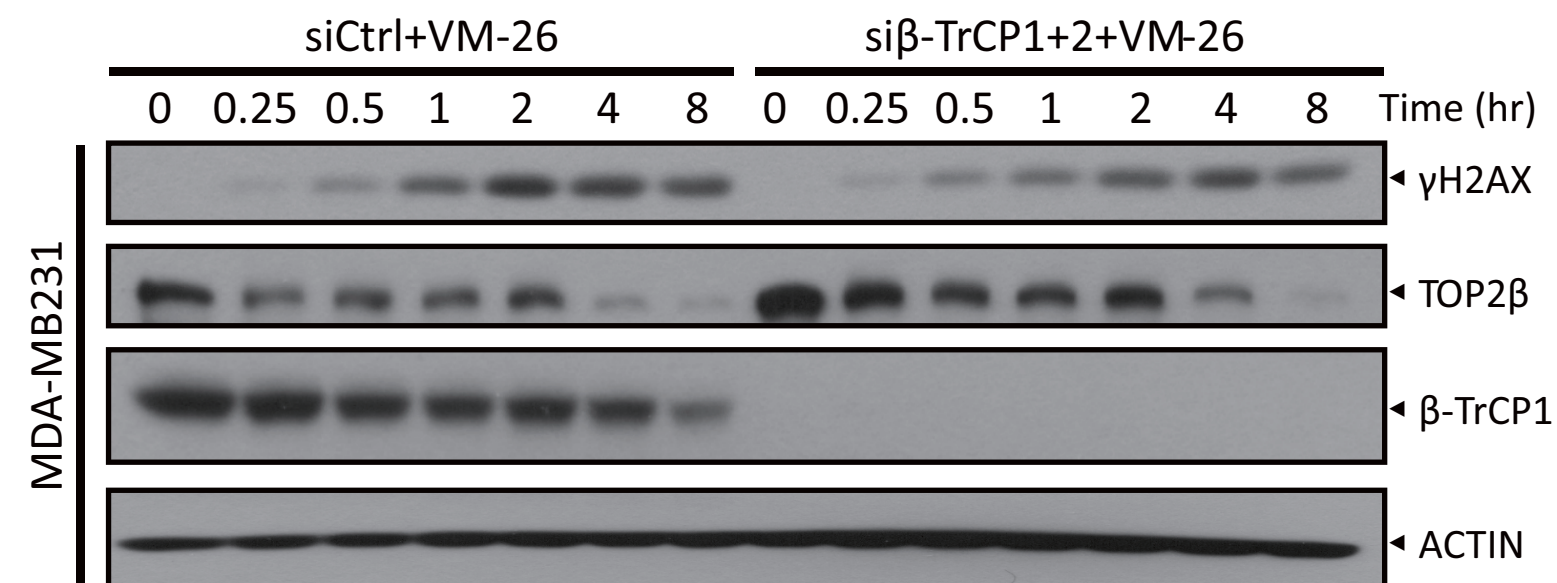

Supplement: Supplementary file 6 — Figure S5 [file 41389_2020_196_MOESM6_ESM.pdf]

A

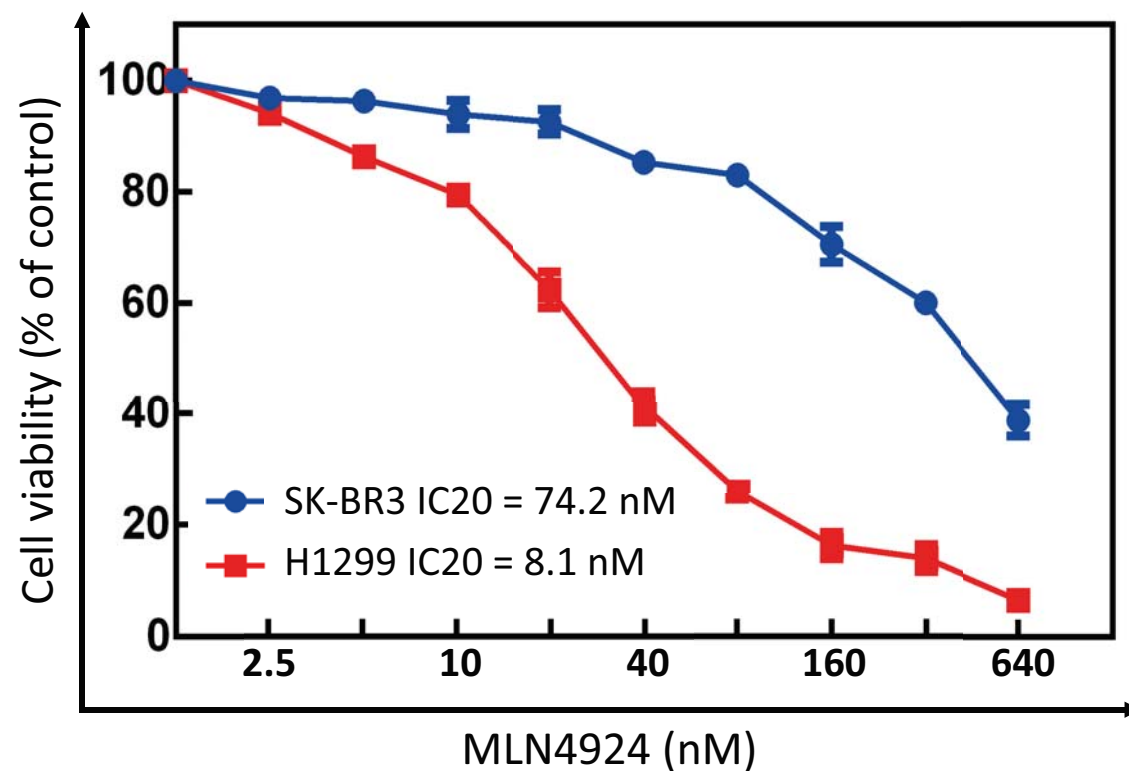

B

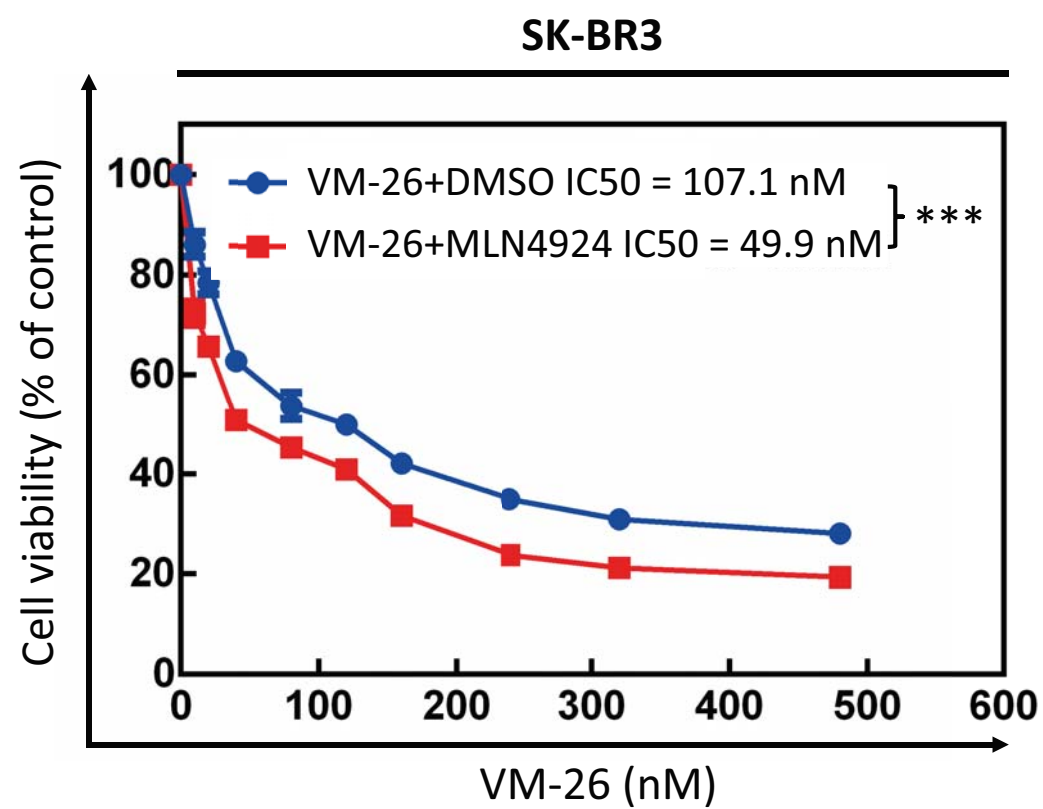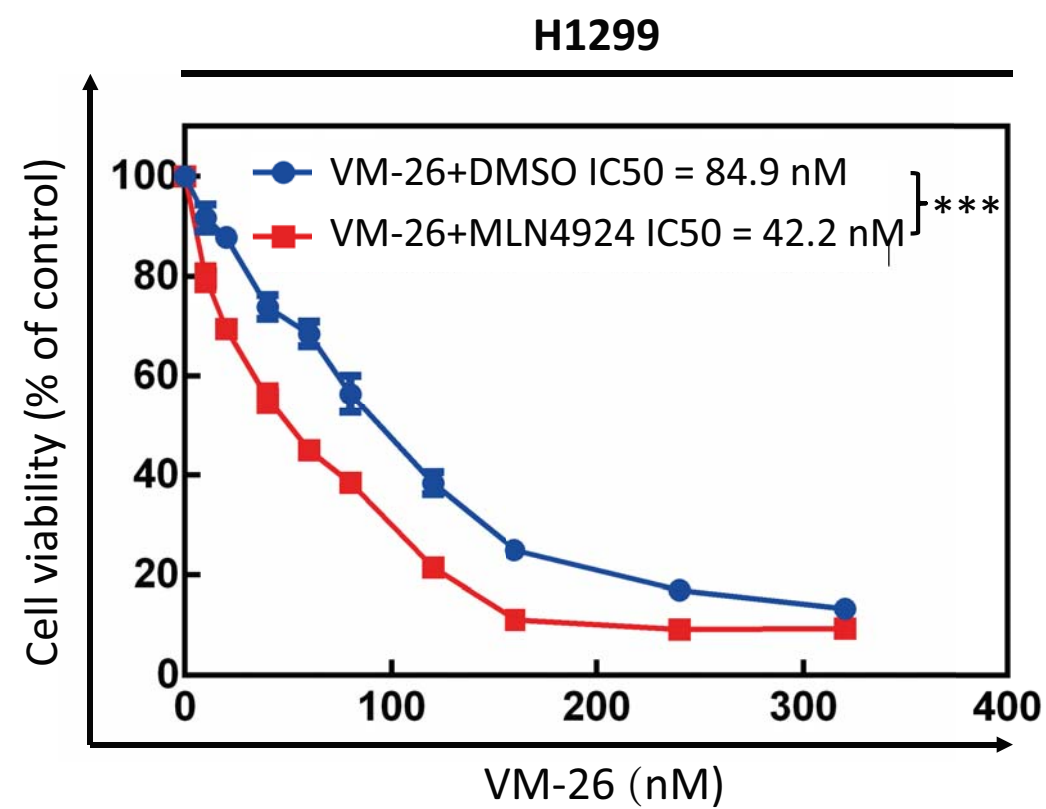

Supplement: Supplementary file 7 — Figure S6 [file 41389_2020_196_MOESM7_ESM.pdf]

A

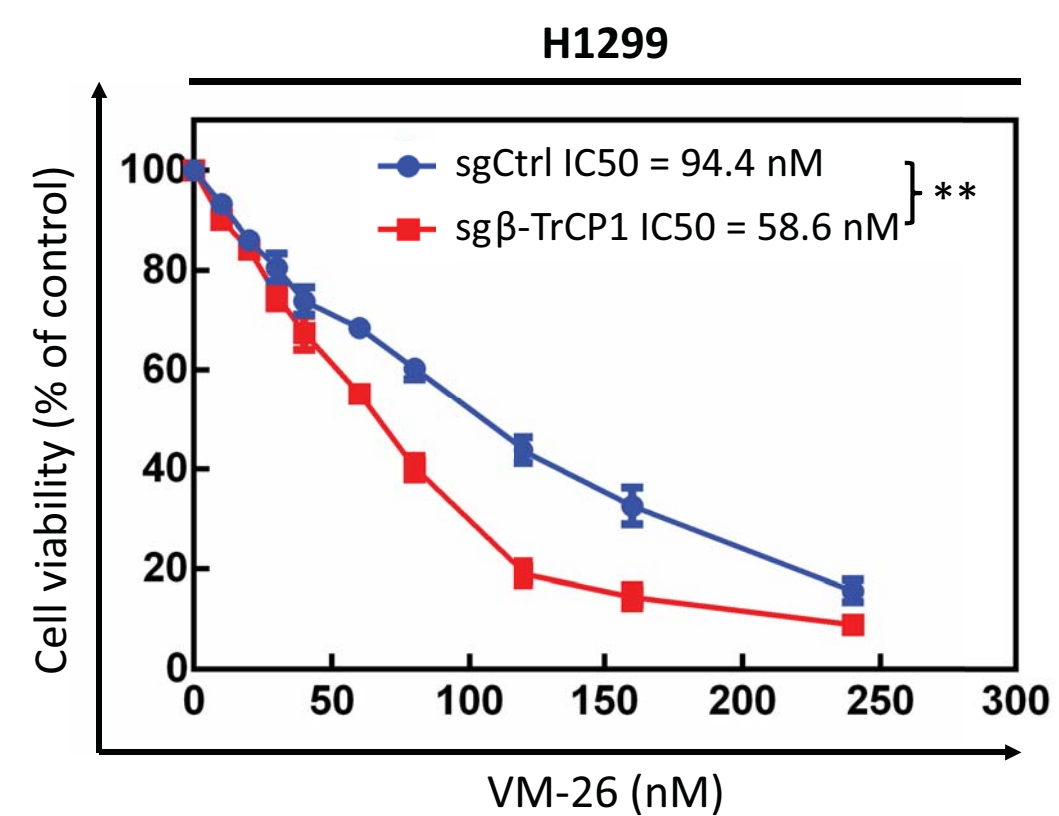

B

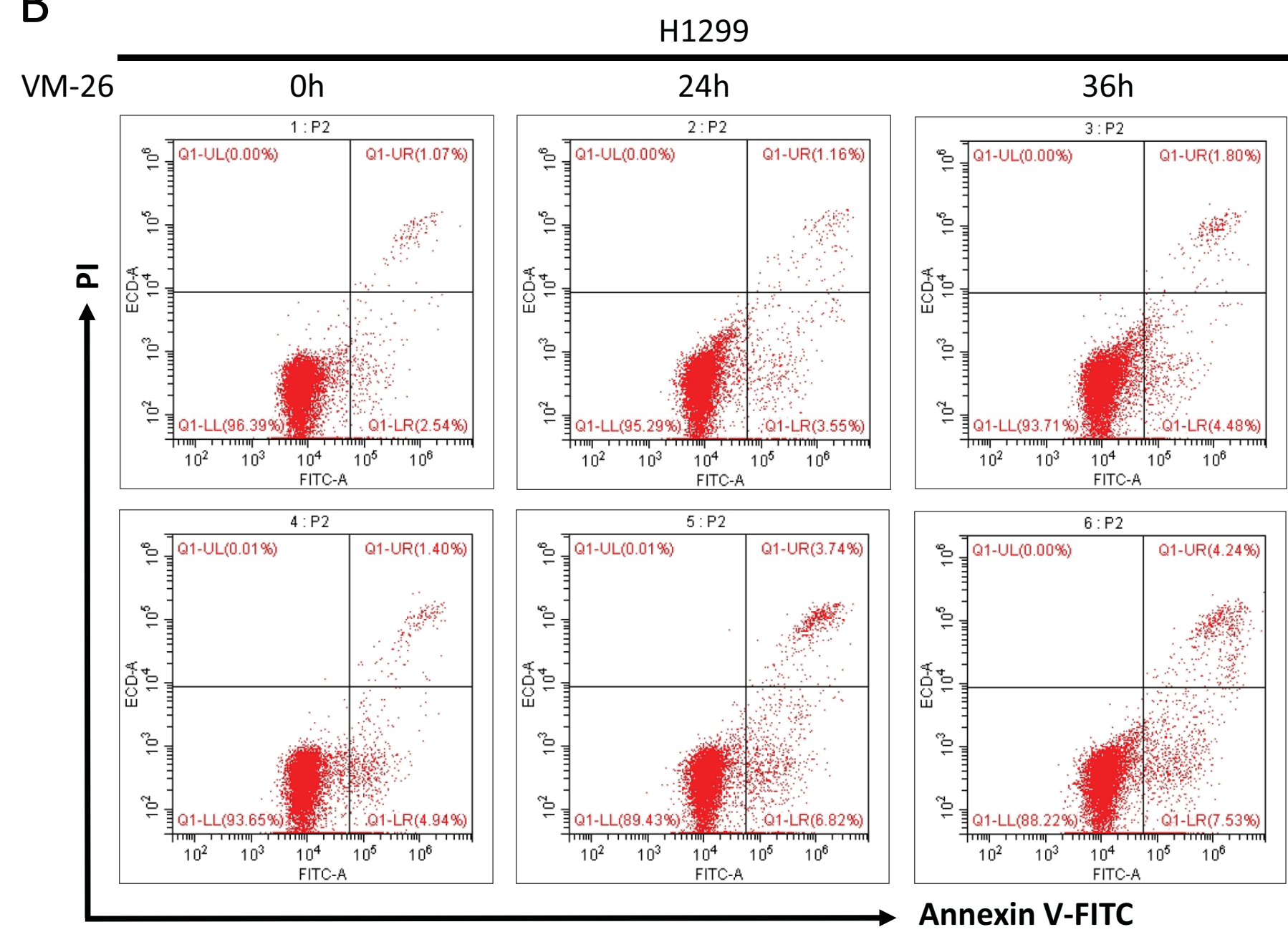

C

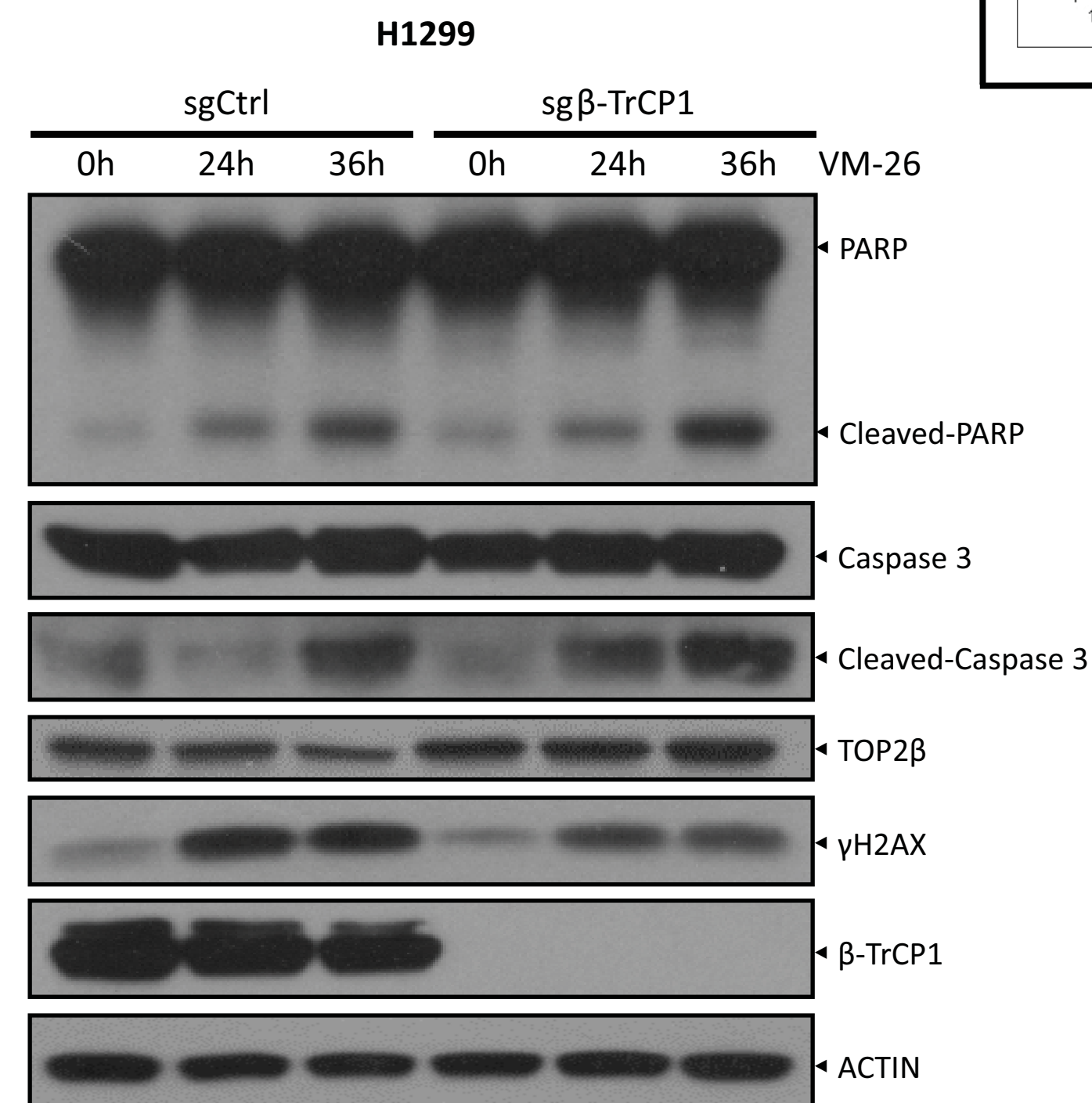

E

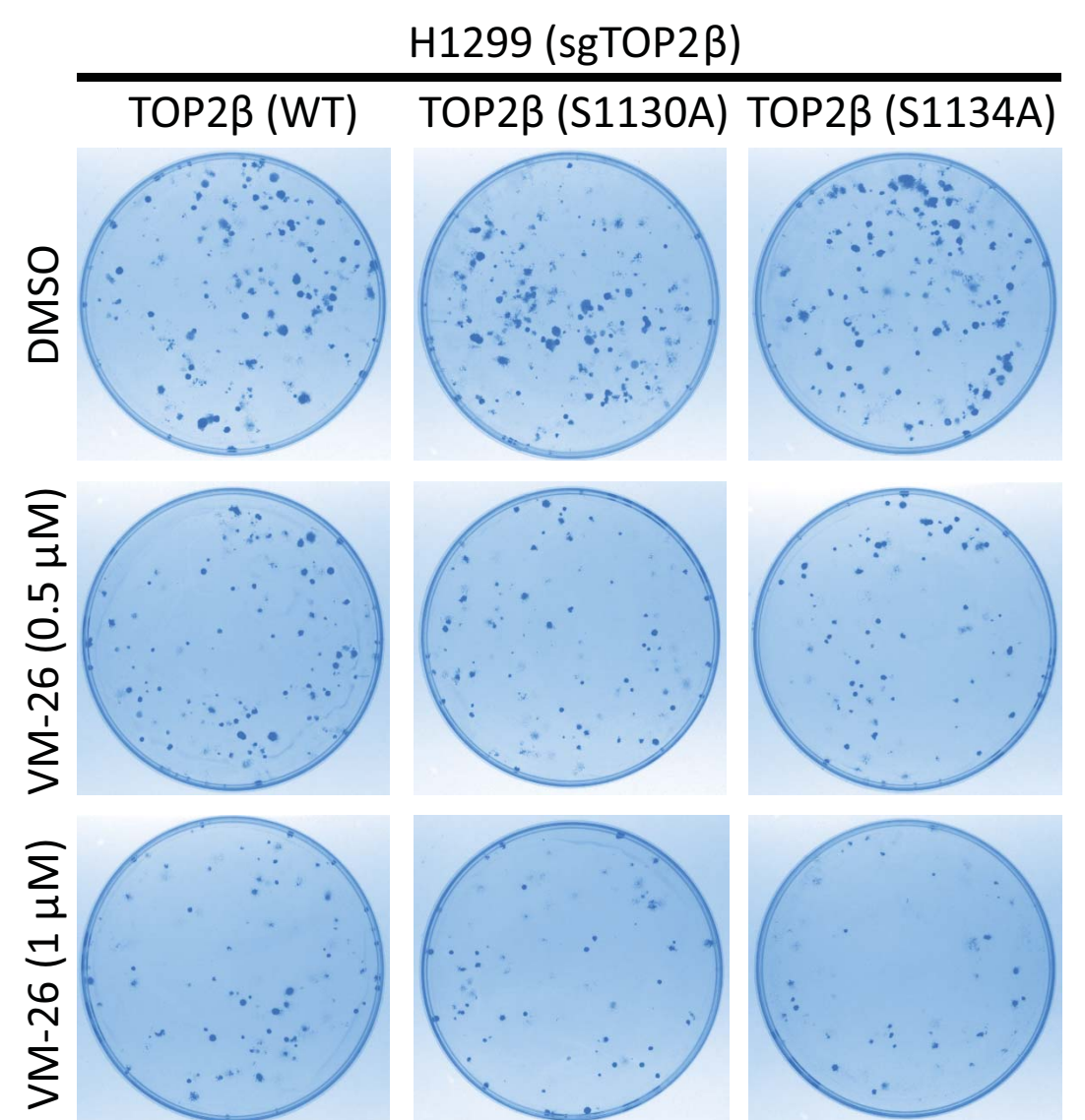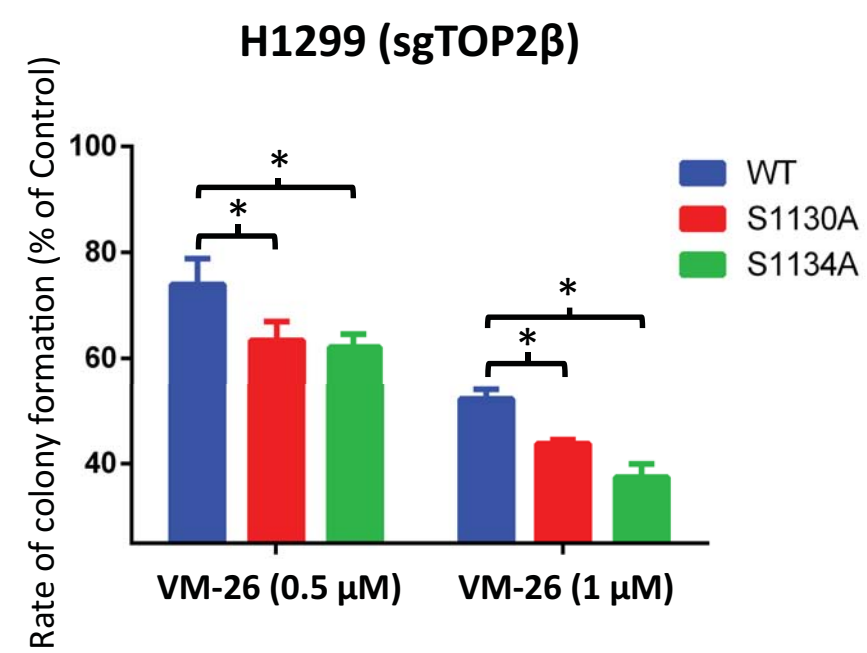

D

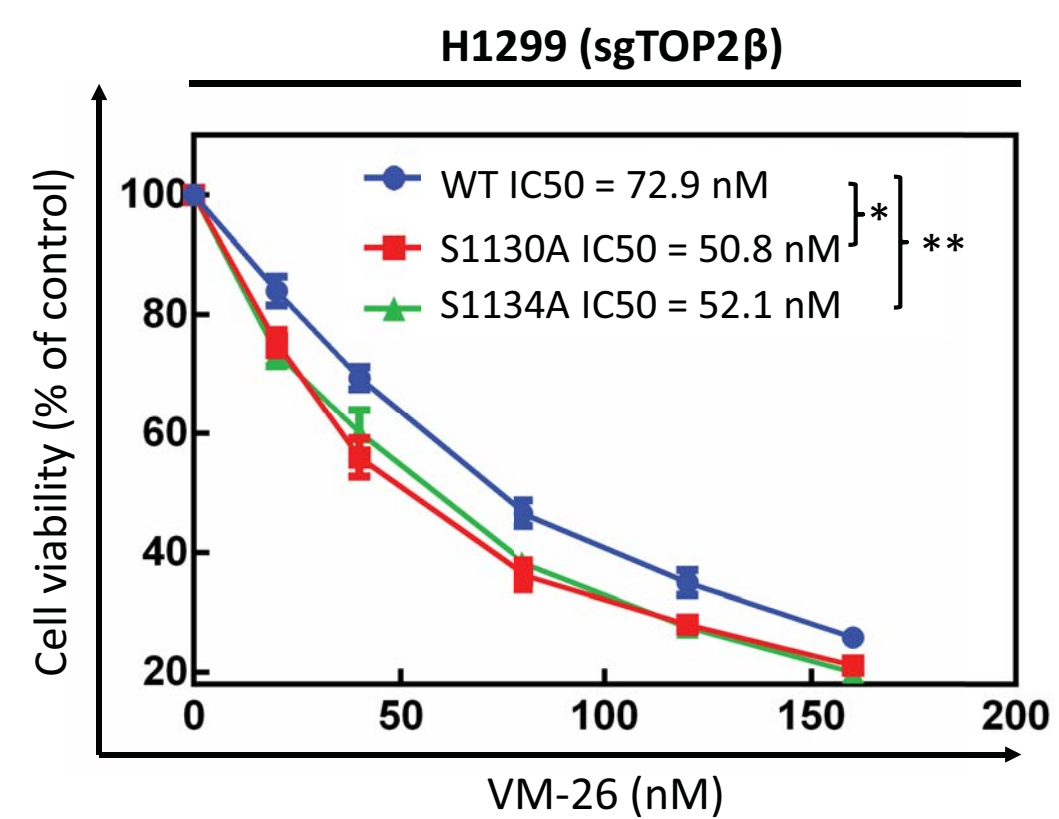

F

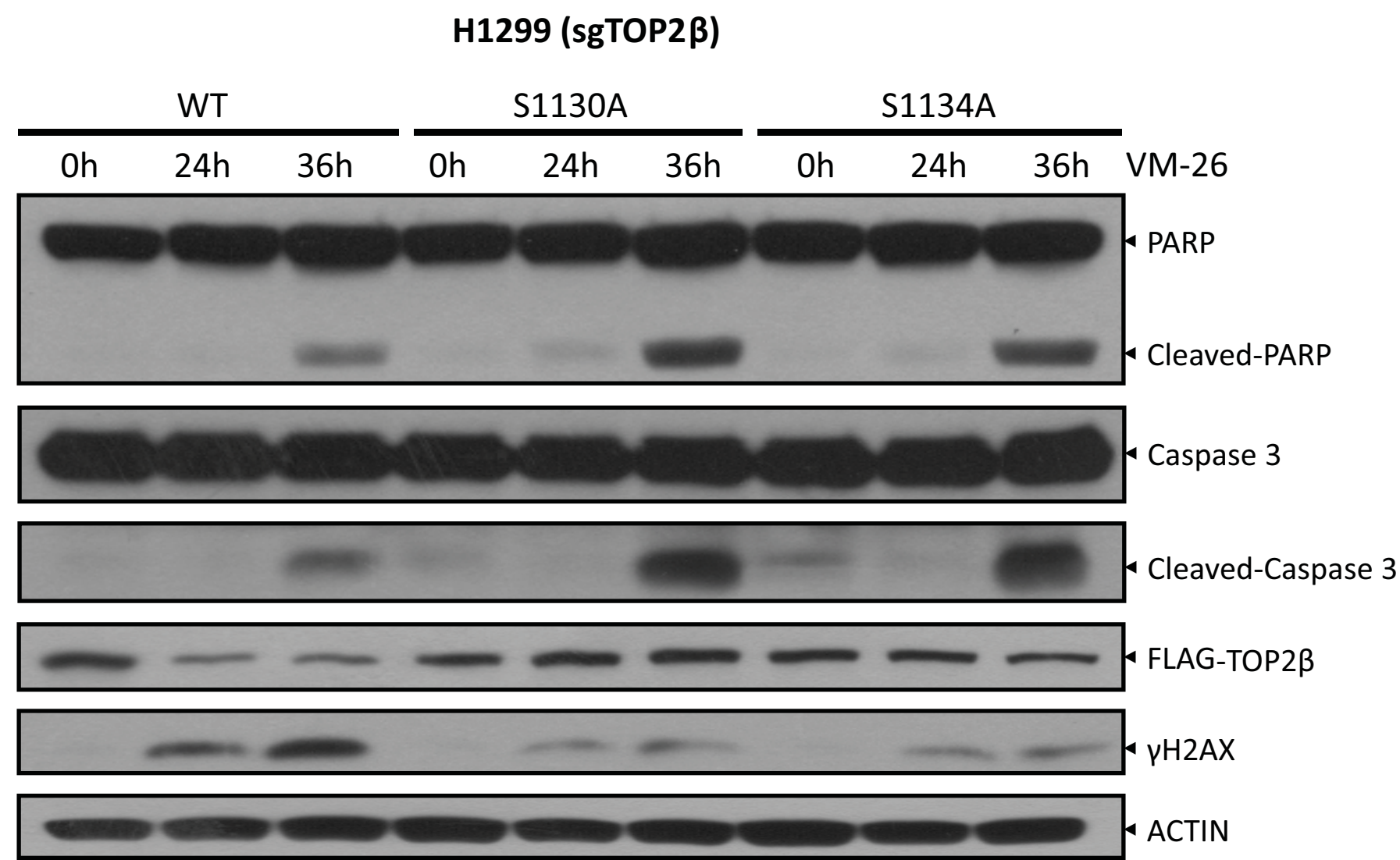

Supplement: Supplementary file 8 — Figure S7 [file 41389_2020_196_MOESM8_ESM.pdf]
